# Tailoring the microstructure, optical, and magnetic characteristics of Co_0.6_Zn_0.4_Fe_2_O_4_ nanoferrites through Ni²⁺–Al³⁺ co-doping

**DOI:** 10.1038/s41598-026-46866-3

**Published:** 2026-05-02

**Authors:** M. Rekaby, M. Ahmed, R. Awad, M. Y. El Sayed, M. A. Abu-Saied, A. I. Abou-Aly

**Affiliations:** 1 Department of Physics, Faculty of Science, Alexandria University, Alexandria, Egypt; 2 Department of Basic Sciences, Faculty of Engineering, Pharos University, Canal El Mahmoudia Street, Alexandria, 21648 Egypt; 3 Department of Basic Sciences, Faculty of Computer Science and Artificial Intelligence, Pharos University, Alexandria, Egypt; 4 Department of Public Health, Faculty of Health Sciences, Modern University for Business and Science, Beirut, Lebanon; 5 Polymer Materials Research Department, Advanced Technologies and New Materials Research Institute, (ATNMRI), City of Scientific Research and Technological Applications (SRTA-City), P.O. Box: 21934, New Borg El-Arab city, Alexandria, Egypt; 6 Faculty of Industrial and Energy Technology, Borg El-Arab Technological University, P.O. Box: 21934, New Borg El- Arab City, Alexandria, Egypt

**Keywords:** Ni^2+^–Al^3+^ co-doping, CoZn-ferrites, UV-vis spectroscopy, VSM, Materials science, Nanoscience and technology, Physics

## Abstract

Herein, Ni²⁺–Al³⁺ co-doped Co_0.6−x_Zn_0.4−x_Ni_x_Al_x_Fe_2_O_4_ (0 ≤ x ≤ 0.08) nanoferrites were synthesized via a co-precipitation route to elucidate the role of defect engineering and cation redistribution in tuning optical magneto-structural properties. X-ray diffraction (XRD) confirmed single-phase cubic spinel formation with slight lattice expansion (8.3667–8.374 Å) accompanied by crystallite size reduction (16.27–11.33 nm). HRTEM and SAED analyses revealed spherical/cubic nanoparticles with high crystallinity and lattice coherence, consistent with XRD results. The increase in microstrain and dislocation density indicates enhanced lattice distortion induced by substitution. XPS analysis revealed mixed Fe²⁺/Fe³⁺ states distributed over tetrahedral and octahedral sites, enabling quantitative cation distribution modeling. The pronounced enhancement of the Raman A_1g_ mode confirms progressive tetrahedral distortion and cation rearrangement. The nanoferrites exhibit soft magnetic behavior with high saturation magnetization (51–58 emu/g) and low coercivity (20–79 Oe). The law of approach to saturation (LAS) analysis reveals a marked decrease in the anisotropy constant, primarily attributed to the dilution of octahedral Co²⁺ ions, reinforced by Al³⁺-induced A–B exchange weakening and nanoscale spin canting. These findings demonstrate that controlled multi-cation substitution provides an effective strategy for tailoring magnetic softness and anisotropy, making the materials promising for spintronics, high-frequency electronics, magnetic sensing, and electromagnetic interference shielding applications.

## Introduction

The soft magnetic characteristics, high electrical resistivity, and chemical stability of spinel ferrites make them a family of materials with significant potential in nanoscience. Effective magnetization and demagnetization are made possible by these features, which are further reinforced by unique optical and electrical behaviors^1^. Because of their exceptional thermal stability and the capacity to modify their properties through cation distribution and synthesis methods, they are a primary focus of advanced materials research^2^. With regard to their adaptability, they may be used in a variety of fields, including electronics, microwave devices, radar absorption, gas sensing, catalysis, ferrofluids, and medical technologies like drug delivery and magnetic hyperthermia cancer therapy^3^. Spinel ferrites typically have the formula MFe_2_O_4_, where Fe is a trivalent iron cation and M represents one or more divalent metal ions, such as Ni^2+^,Co^2+^, Mg^2+^, or Zn^2+^^4^.The deepest anion sites in the spinel ferrite structure are occupied by oxygen ions. On the other hand, in a face-centered cubic lattice, metal cations occupy one-eighth of the tetrahedral (A) and octahedral (B) sites^5^.

Spinel ferrites are categorized based on the distribution of cations between A and B sites. Normal, inverse, and mixed are the three types of spinel ferrites^6,7^. A normal spinel ferrite is distinguished by the full occupancy of B sites by a single kind of cation. ZnFe_2_O_4_ and AlFe_2_O_4_, for example, have normal spinel structures with nanoscale structural changes that might lead to a partly inverse spinel^8^. Because of their chemical and thermal stability as well as the fact that their magnetic properties depend on particle sizes, spinel ferrites have been extensively studied^9^. In an inverse spinel ferrite, half of the Fe^3+^ ions are located in the A sites, whereas the remaining Fe^3+^ and divalent metal ions (M^2+^) are distributed among the B sites^10^. One well-known example of partial cationic inversion is cobalt ferrite (CoFe_2_O_4_). The distribution of Fe^3+^ and Co^2+^ is determined using the synthesis technique. CoFe_2_O_4_ nanoparticles are particularly fascinating due to their low band gap energy (2.04 eV), strong saturation magnetization, mechanical hardness, chemical stability, visible light absorption, and mild ferromagnetic properties^11,12^. Another inverse spinel, nickel ferrite (NiFe_2_O_4_), undergoes a shift to a mixed spinel configuration at the nanoscale, where its characteristics are affected by the size, shape, and synthesis circumstances of the particles^13^. Ferrites with a variety of particle sizes and shapes may be produced using different production processes. Solid-state^14^, co-precipitation^15^, combustion^16^, oxidation^17^, sol-gel synthesis^18^, microwave hydrothermal synthesis^19,20^, and hydrothermal processes^21^ are some well-known methods. The co-precipitation approach is superior due to its controlled microstructure, cost, convenience of application, and efficiency. The numerous uses of cobalt (CoFe_2_O_4_) and zinc (ZnFe_2_O_4_) ferrites captured the curiosity of researchers^12–15^.

Numerous research studies have examined the impact of substituting other metals (Zn^2+^, Cu^2+^, and Ni^2+^) for Co^2+^ in CoFe_2_O_4_ ferrite on the ferrite’s structural and magnetic properties^22–25^. When non-magnetic Zn^2+^ ions are substituted for ferromagnetic Co^2+^ ions, cobalt-zinc ferrites (CoZn-ferrites) are created. This dilution causes cation redistribution between A and B sites, which drastically changes the structural and magnetic characteristics^26^. Due to its soft magnetic nature, modest saturation magnetization (M_s_), high resistivity, and low eddy current losses, CoZn-ferrites are much sought after in applications such as sensors and high-frequency inductive components^27,28^. Akhtar et al.^28^ studied CoZn-ferrites with Nd–Cu co-doped replacements with the formula Co_0.5_Zn_0.5−x_Cu_x_Fe_2−y_Nd_y_O_4_, where x = 0.0, 0.1, 0.2, 0.3, 0.4, 0.5, and y = 0.00, 0.02, 0.04, 0.06, 0.08, 0.10.

After Nd–Cu co-doping, the CoZn-ferrites’ structural, magnetic, and dielectric characteristics altered significantly. The more homogeneous morphology of the undoped samples was replaced by the formation of diverse grain morphologies (elliptical, circular, and hexagonal). Rietveld refinement verified small changes in the cation distribution and lattice properties. The magnetization and coercivity steadily reduced as the Nd–Cu concentration rose, suggesting a modified switching field and improved high-frequency responsiveness. Additionally, the dielectric permittivity and permeability increased at high frequencies due to increasing interfacial polarization. Bi-Sn co-doped CoZn ferrite nanoparticles, Co_0.5− x_Zn_0.5−x_BiₓSnₓFe₂O₄ (0.00 ≤ x ≤ 0.10), were created by co-precipitation^29^. The structural study confirmed the formation of spherical nanoparticles with a mostly single-phase spinel structure. Co-doping changed the optical band gaps and recombination rates, as demonstrated by the UV-vis and PL studies. The successful substitution of Bi³⁺ and Sn²⁺ for Co²⁺ and Zn²⁺ was verified by EDX spectroscopy. Co_1−x_ZnₓFe_2−x_AlₓO₄ (0 ≤ x ≤ 0.15) ferrites synthesized by glycine–nitrate auto-combustion were studied by Shashanka et al.^30^. Since Al³⁺ ions replaced bigger Fe³⁺ ions, both as-prepared and sintered samples maintained the cubic spinel phase, with crystallite size and lattice parameter dropping as x rose. Al–Zn co-doping altered metal–oxygen bond lengths, moved tetrahedral and octahedral IR/Raman bands to higher wavenumbers, and promoted grain development into dense, interconnected morphologies (SEM). Zn²⁺ inhabited tetrahedral sites, whereas Al³⁺ occupied octahedral sites, according to Mössbauer analysis. Curie temperature (T_c_), magnetocrystalline anisotropy (K_1_), and coercivity (H_c_) all dropped with x as a result of the nonmagnetic ions’ reduced A–O–B superexchange. Due to equivalent magnetic dilution at both locations, the saturation magnetization (Ms) remained similar to pure CoFe₂O₄.

Almousawi et al.^9^ described the synthesis of Co_0.3_Ni_0.3_Cd_0.2_Cu_0.2_Fe_1.8_Ce_0.2−x_Sb_x_O_4_ (0 < x < 0.12) ferrites using the co-precipitation technique. XRD indicated a dominating spinel phase with a minor hematite phase. TEM and SEM revealed rounded-edged cubic particles. EDX revealed a homogenous elemental distribution, but XPS revealed multivalent Ce with Sb³⁺ instead of Fe³⁺. Weak ferromagnetism was indicated by the small hysteresis loops seen in VSM tests. The impact of Al^3+^ doping in nickel-zinc-cobalt (Ni–Zn–Co) mixed spinel ferrites was examined in a few articles. For example, Jahan et al.^31^ produced Al^3+^ doped Ni_0.4_Zn_0.35_Co_0.25_Fe_2−x_Al_x_O_4_ (0 ≤ x ≤ 0.12) using the traditional ceramic method. XRD and SAED verified the samples’ crystallinity, with particle sizes ranging from 0.39 to 0.67 μm.

Additionally, co-doping cobalt ferrite with Ce³⁺ and Mn²⁺ ions, Co₂₋ₓMnₓFe₂₋ₓCeₓO₄, may significantly improve its magnetic performance, according to recent research^32^. Cation substitution and related changes in crystallite size are responsible for these advances. Similarly, co-doping cobalt ferrite with Zn²⁺ and Ti²⁺, CoFe₂₋₂ₓZnₓTiₓO₄, has been shown by Rajenimbalkar et al.^33^ to alter its structural, magnetic, electrical, and dielectric characteristics. The nanoparticles produced via sol-gel auto-combustion showed soft ferrimagnetic behavior with decreased coercivity, increased resistivity, and frequency-dependent dielectric response while maintaining a single-phase cubic spinel structure with nanoscale spherical grains. Furthermore, cobalt ferrite nanostructures have been modified by Mg²⁺–Ce³⁺ co-doping, according to Suryanarayana et al.^34^. XRD confirmed that the Co_1−x_Mg_x_Fe_2−y_Ce_y_O_4_ (x = 0.0–0.75; y = 0.0–0.09) nanoparticles had aggregated, almost spherical morphologies and single-phase spinel structures. They came to the conclusion that Mg²⁺–Ce³⁺ co-doping effectively modifies CoFe₂O₄ nanoferrites’ electrical and magnetic properties.

The influence of co-doping Al^3+^ and Ni^2+^ on Co^2+^ and Zn^2+^ sites of CoZn-ferrites has not yet been studied. This work presents a unique method of simultaneously co-doping Ni^2+^ and Al^3+^ to modify the characteristics of CoZn-ferrites. Controlled cation substitution may drastically change the structural and magnetic characteristics of CoZn-ferrite nanoparticles, which are well recognized for their wide range of scientific and technological applications. In order to maintain the spinel structure and prevent phase change, CoZn-ferrite nanoparticles with the composition Co_0.6−x_Zn_0.4−x_Ni_x_Al_x_Fe_2_O_4_, at doping levels of Ni^2+^and Al^3+^ x = 0.00, 0.01, 0.02, 0.04, and 0.08. It is anticipated that the co-doping of Ni^2+^ and Al^3+^ will have a synergistic impact, optimizing the material’s structural and magnetic characteristics and preparing it for cutting-edge uses such as microwave devices, sensors, and catalysis. XRD, HRTEM, selected area electron diffraction (SAED), SEM, EDX, UV-vis spectroscopy, FTIR, Raman spectroscopy, XPS, and VSM measurements are used to examine the produced materials’ structural, morphological, optical, and magnetic characteristics. By providing new insights into the complementing effects of Ni²⁺ and Al³⁺ co-doping, these investigations are expected to aid in the design of high-performance CoZn-ferrites.

## Experimental technique

### Materials and synthesis

**Fig. 1:**
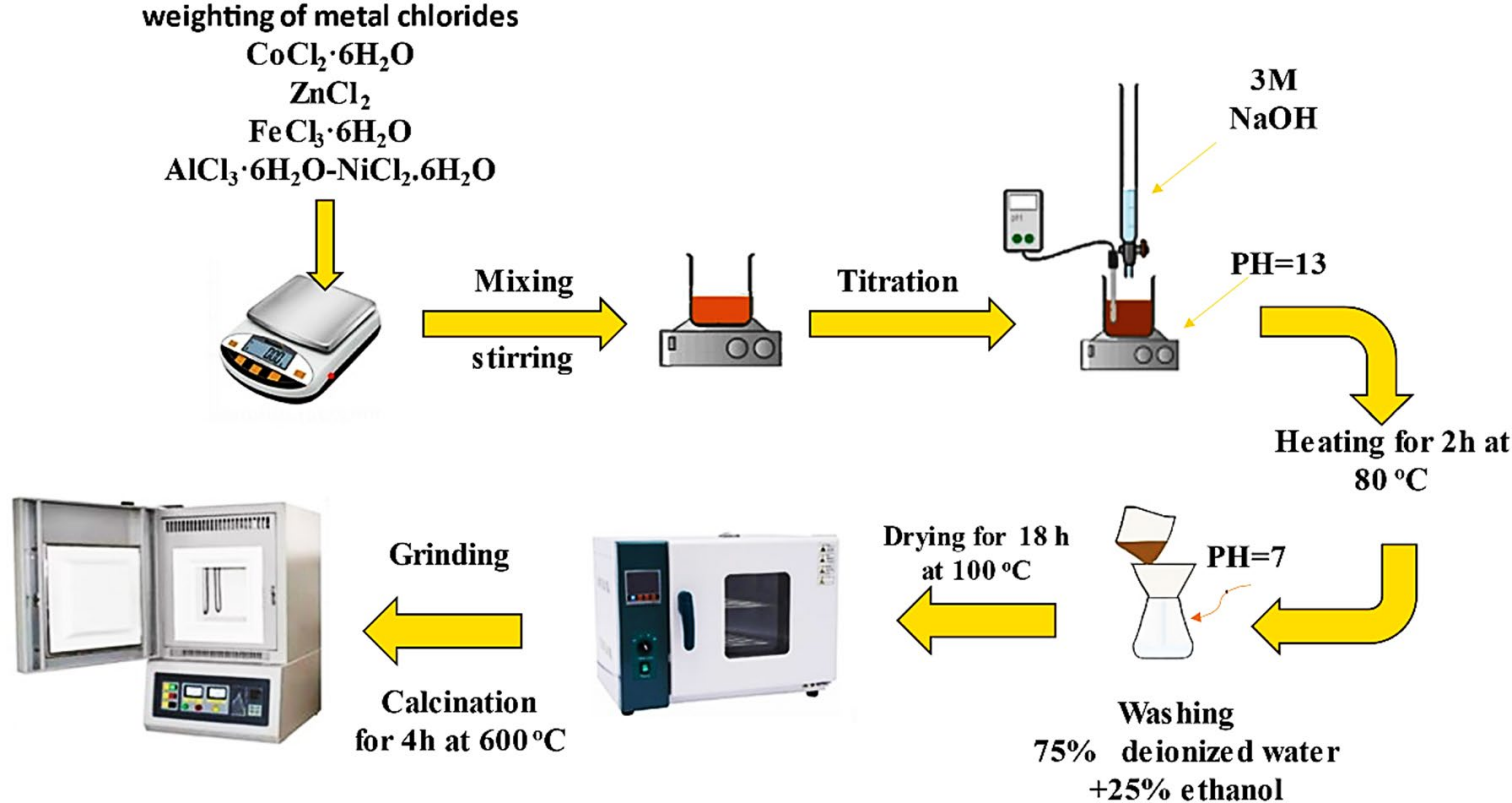
Schematic illustration of the co-precipitation synthesis procedure for Co₀.₆₋ₓZn₀.₄₋ₓNiₓAlₓFe₂O₄ nanoparticles.

The co-precipitation approach^9^ was used to create Co_0.6−x_Zn_0.4−x_Ni_x_Al_x_Fe_2_O_4_ nanoparticles with x = 0.00, 0.01, 0.02, 0.04, 0.06, and 0.08. Cobalt chloride hexahydrate (CoCl_2_·6H_2_O, ≥ 97%, Loba Chemie), zinc chloride (ZnCl_2_ anhydrous, > 98%, Sigma Aldrich), and ferric chloride hexahydrate (FeCl_3_·6H_2_O, ≥ 97%, Oxford) were the starting materials for the pure Co_0.6_Zn_0.4_Fe_2_O_4_ sample. After dissolving stoichiometric proportions of the starting materials in distilled water, the mixture was magnetically stirred for 20 min. The solutions were then titrated with 3 M NaOH to reach a pH of 13. After that, the solutions were heated to 80 °C while being continuously stirred magnetically to create a highly viscous gel. After two hours, the mixtures were allowed to cool to ambient temperature for 30 min. To get a pH of 7, the produced combinations were washed with a solution of 75% distilled water and 25% ethanol. The precipitates were dried for 18 h at 80 °C. The dried precipitates were manually crushed for 10–15 min to create a fine and uniform powder for annealing. The powder was then heated at 600◦C for 4 h. To co-dope Co_0.6_Zn_0.4_Fe_2_O_4_ samples with Ni²⁺ and Al³⁺, the same technique was followed with the appropriate quantities of aluminum chloride hexahydrate (AlCl_3_·6H_2_O, ≥ 98%, Qualikems) and nickel chloride hexahydrate (NiCl_2_.6H_2_O, ≥ 97%, Oxford) added to the chloride mixture precursors to get Co_0.6−x_Zn_0.4−x_Ni_x_Al_x_Fe_2_O_4_, with x = 0.01, 0.02, 0.04, 0.06, and 0.08. Figure 1 shows a schematic diagram of the preparation stages.

### Characterization and measurements

The structure of the generated nanoparticles (NPs) was examined by XRD using a Bruker D8 Advance equipment with Cu-Kα radiation (λ = 1.5406 Å). The recorded diffraction patterns were refined using the Rietveld approach with material analysis employing diffraction (MAUD) software and CIF files from the Crystallography Open Database. The morphology, agglomeration, and particle size distribution were investigated using a Transmission Electron Microscope (TEM) (JOEL, JEM-2100, Tokyo, Japan) at an accelerating voltage of 200 kV. ImageJ software was employed to measure particle sizes from TEM micrographs. Lattice planes and crystallinity were further examined using HRTEM. SEM combined with EDX spectroscopy using a JEOL JSM-IT200 device was used to assess the grain shape and elemental composition. The U-2900 spectrophotometer was used to record the UV–vis spectra at room temperature in the wavelength range of 300–550 nm. A Bruker Vertex 70-Germany-KBr spectrometer was used to capture FTIR spectra in the wavenumber range of 4000–350 cm^− 1^. An Alpha 300RS WITec Raman confocal microscope was used to perform Raman spectroscopy at room temperature. A 785 nm laser excitation source, a 50X magnification objective, and a power of around 5 mW were used to produce the Raman spectra prior to analysis. A K-Alpha (ThermoFisher Scientific, USA) device with monochromatic Al-Kα radiation (energy range − 10 to 1350 eV, spot size 400 μm, pressure 10^− 9^ mbar) was used for the XPS experiments. XPS spectra were recorded at different spots on the sample surface to confirm consistency in Fe²⁺/Fe³⁺ ratios and peak deconvolution. A Lake Shore VSM 7410 system with a magnetic field range of 20 kG to 20 kG was used to analyze the samples’ magnetic characteristics at room temperature. VSM were performed multiple times for each composition, and the variation in Mₛ and H_c_ values was within experimental uncertainty (± 2–3%). Curve fitting of the magnetic hysteresis loops using several models in Wolfram Mathematica allowed for accurate identification of the pertinent magnetic characteristics.

## Results and discussion

### Structural/microstructural analysis

#### XRD

**Fig. 2:**
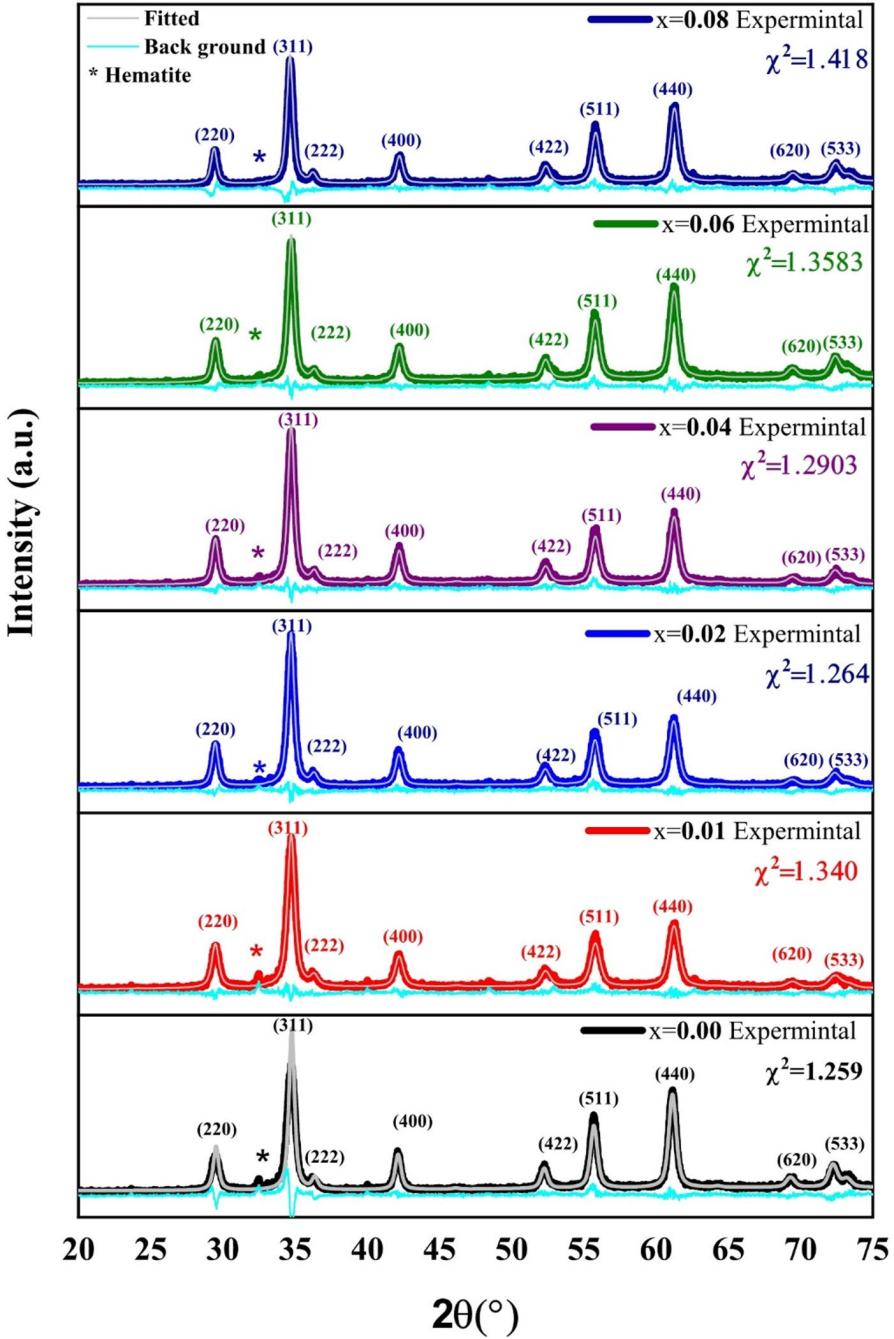
XRD patterns and rietveld refinement of Co_0.6−x_Zn_0.4−x_Ni_x_Al_x_Fe_2_O_4_ with x = 0.00, 0.01, 0.02, 0.04, 0.06, and 0.08.

**Fig. 3:**
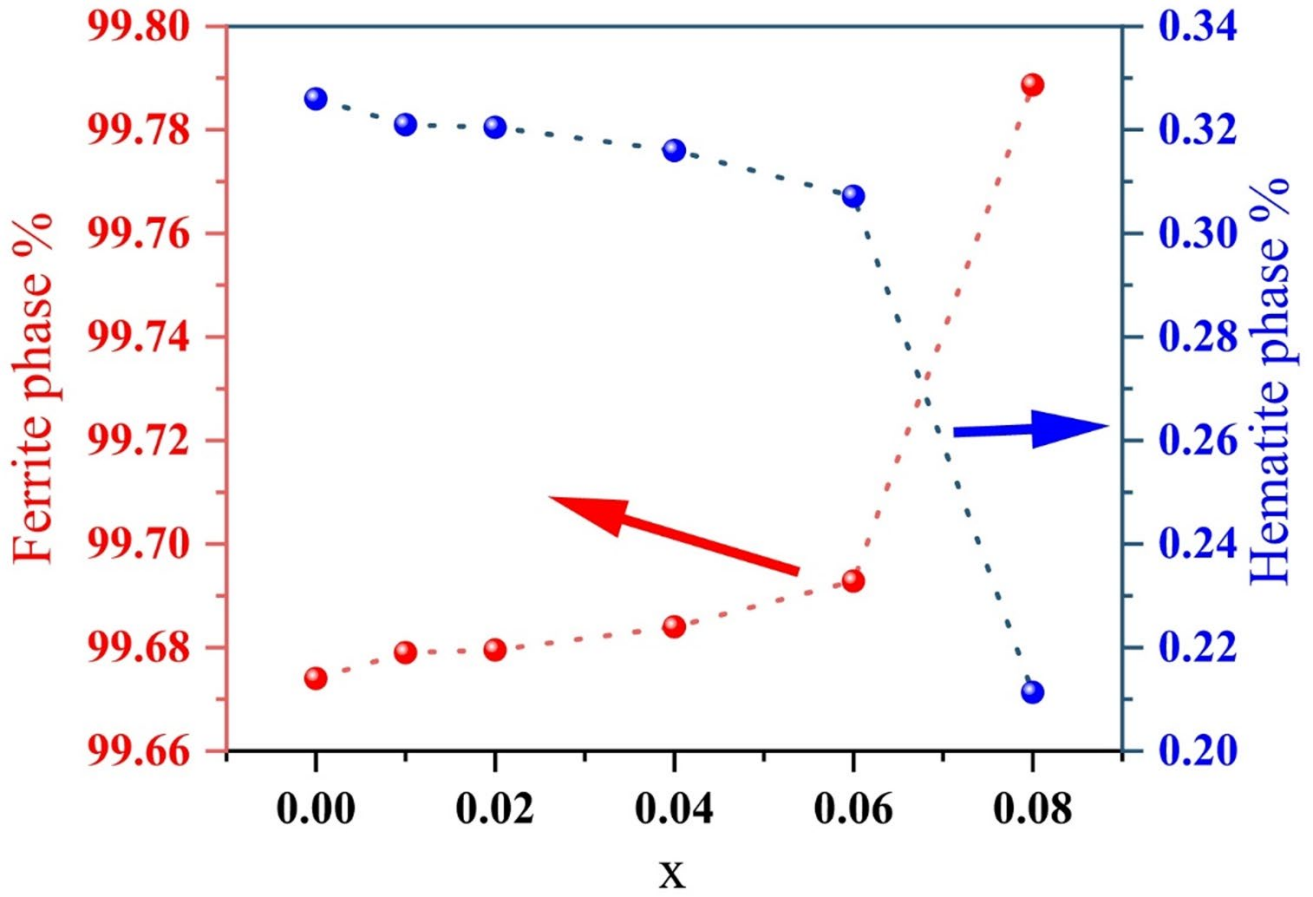
Variation of primary ferrite and secondary hematite (α-Fe₂O₃) phase percentages as a function of Ni²⁺–Al³⁺ co-doping concentration (x), showing the suppression of the hematite phase with increasing dopant content.

The XRD patterns were fitted using the MAUD software, which is based on the Rietveld refinement process. Figure 2 shows the experimental and fitted XRD patterns of Co_0.6−x_Zn_0.4−x_ Ni_x_Al_x_Fe_2_O_4_ (0.00 ≤ x ≤ 0.08), confirming the material’s structure and purity. Nine major peaks were identified at 2θ values of approximately 30.2°, 35.54°, 37.19°, 43.13°, 53.58°, 57.10°, 62.68°, 73.71°, and 75.23°. The corresponding planes are as follows: (220), (311), (222), (400), (422), (511), (440), (533), and (620). These patterns are compatible with the cubic spinel ferrite structure with the Fd$\:\stackrel{-}{3}$m space group as described in (JCPDS No. 22–1086)^35^. As shown in Fig. 2, a small peak at 33.21° indicates the presence of hematite (α-Fe₂O₃) as a secondary phase. This may be due to incomplete incorporation of Fe³⁺ into the spinel lattice as a result of cation imbalance^36^. Figure 3 illustrates that the percentage of hematite is very low in the x = 0.00 sample (about 0.32%) and decreases to 0.21% at x = 0.08. The small amount of hematite confirms the high purity of the synthesized samples. This reduction suggests that Ni–Al co-doping effectively suppresses hematite formation^37^. Similar results have been reported in previous studies^38^.

Using the interplanar distance (d) and Miller indices (hkl), the lattice parameter (a) of the synthesized nano-ferrites was calculated as follows^39^:

1 $$\:\mathrm{a}=\mathrm{d}\sqrt{{\mathrm{h}}^{\mathrm{2}}\mathrm{+}{\mathrm{k}}^{\mathrm{2}}\mathrm{+}{\mathrm{l}}^{\mathrm{2}}}.$$

The obtained values for lattice parameter (a) versus Ni²⁺–Al³⁺ content (x) were given in Table 1. It is clear, from Table 1, that as the concentration of Ni²⁺–Al³⁺ content raises from 0.00 to 0.08, the lattice parameter slightly increases from 8.3667 Å to 8.374 Å. The lattice parameter (a) is expected to decrease with Ni²⁺–Al³⁺ co-doping because Al³⁺ (≈ 0.535 Å) and Ni²⁺ (≈ 0.69 Å) have smaller ionic radii than Co²⁺ (≈ 0.745 Å) and Zn²⁺ (≈ 0.74 Å) in octahedral coordination. However, the valence mismatch between the dopant ions (Ni²⁺, Al³⁺) and the host cations (Co²⁺, Zn²⁺) disturbs the charge balance of the lattice. To maintain charge neutrality, some Fe³⁺ ions (0.64 Å, octahedral site) are reduced to Fe²⁺ (0.78 Å, octahedral site)^40^. Since Fe²⁺ has a larger ionic radius than Fe³⁺, this conversion results in lattice expansion and an increase in the lattice parameter. This valence change will be confirmed later by XPS analysis. As shown in Fig. 2, the main (311) diffraction peak shifts toward lower 2θ values, indicating an increase in interplanar spacing and lattice parameter according to Bragg’s law^12^. Similar behavior was reported by Kaur et al.^12^ for Cr-doped CoFe₂O₄, and by Chahar et al.^36^ for Ni-substituted Co–Zn ferrites.

The crystallite sizes (D_DS_) were calculated from Debye-Scherrer^41–43^ according to the following formula:

2 $$D_{DS} = \frac{k\lambda}{\beta \cos \theta}$$

where k is the geometric factor for spherical particles equals 0.9, β is the full width at half maximum (FWHM), θ is the diffraction angle, and λ is the wavelength of x-ray (1.5406 Å) radiation. It is evident from Table 1 that as the Ni^2+^-Al^3+^ content rises from 0.00 to 0.08, the crystallite size, D_DS_, falls from 16.267 nm to 11.325 nm. The observed increase in FWHM and decrease in peak intensity, as shown in Fig. 2, which indicate a decrease in crystallinity and crystallite size, support this trend. Even while smaller crystallites are frequently the result of lattice disorder, the consistent reduction in crystallite size with increasing Ni^⁺2^ -Al^+ 3^ composition lends credence to the notion of more lattice distortion and restricted grain formation as a result of the dopant-induced strain^38^. The following equation is used to calculate the microstrain (ε)^39^:

3 $$\:\epsilon\:=\frac{\beta\:}{4tan\theta\:\:}\:\:$$.

Table 1 makes it clear that when the Ni^2+^-Al^3+^ content rose from x = 0.00 to x = 0.08, the microstrain values increased from 2.26 × 10⁻³ to 3.24 × 10⁻³. This trend suggests that Ni^2+^-Al^3+^ substitution introduces significant lattice distortion because its ionic radius is smaller than that of Co^2+^ and Zn^2+^. The local stress brought on by the mismatch in ionic radii inside the crystal structure increases the internal strain. The dislocation density (δ) is computed through the following equation^39^:

4 $$\:{\updelta\:}=\frac{1}{{{D}_{DS}}^{2}}$$

The number of crystal flaws is indicated by the dislocation density. The findings in Table 1 demonstrate that when x increases from 0.00 to 0.08, the dislocation density δ values increase from 3.778 × 10^− 3^ to 7.795 × 10^− 3^. As Ni^2+^-Al^3+^ increased, more defects, including oxygen vacancies, were produced. The enhancements in microstrain (ε) and dislocation density (δ) weaken the A–B superexchange interactions and facilitate spin canting, particularly at particle surfaces^39^. The X-ray density ρ_x_ was determined using the following equation^39^:

5 $$\:{{\uprho\:}}_{\mathrm{x}}=\frac{8\:\mathrm{M}}{{\mathrm{N}}_{\mathrm{A}}{\mathrm{a}}^{3}}$$

where M is the molecular weight and N_A_ is Avogadro’s number. Table 1 illustrates that the X-ray density, ρ_x_, increases from 5.38 to 5.495 g.cm^− 3^ with the Ni^+ 2^-Al^+ 3^ co-doping. The specific surface area (S) is determined using the following expression^39^:

6 $$\:\mathrm{S}=\frac{6000}{{{\uprho\:}}_{\mathrm{x}}{\mathrm{D}}_{DS}}.$$

It is noticeable from Table 1 that the specific surface area values rise from 6.8555 m^2^ g^− 1^ for x = 0.00 to 9.639 m^2^ g^− 1^ for x = 0.08. The enhancement in S is a direct consequence of the observed crystallite size decrement with Ni^+ 2^-Al^+ 3^ doping. This pattern, which has already been noted in the literature^42^, suggests that crystallite size and specific surface area are inversely related.

**Table 1:** Structural and microstructural parameters of Co₀.₆₋ₓZn₀.₄₋ₓNiₓAlₓFe₂O₄ nanoparticles, including lattice parameter (a), crystallite size (D_DS_), unit cell volume (V), lattice strain (ε), dislocation density (δ), X-ray density (ρ_x_), specific surface area (S), and selected area electron diffraction (SAED) lattice parameter.

x	a (Å)	D_DS_ (nm)	V(Å^3^)	ε × 10^− 3^	δ × 10^− 3^	ρ_x_ (gm/cm^3^)	S(m^2^ g^−1^)	a_SAED_
0.00	8.3667	16.267	5.857	2.26	3.77	5.38	6.85	8.371
0.01	8.3668	14.375	5.858	2.58	4.83	5.374	7.76	8.374
0.02	8.3681	13.869	5.859	2.65	5.19	5.391	8.02	8.376
0.04	8.3719	13.320	5.867	2.66	5.63	5.422	8.30	8.379
0.06	8.3731	12.691	5.870	2.66	6.20	5.459	8.65	8.383
0.08	8.374	11.325	5.872	3.24	7.79	5.495	9.63	8.387

#### TEM, HRTEM and SAED analysis

**Fig. 4:**
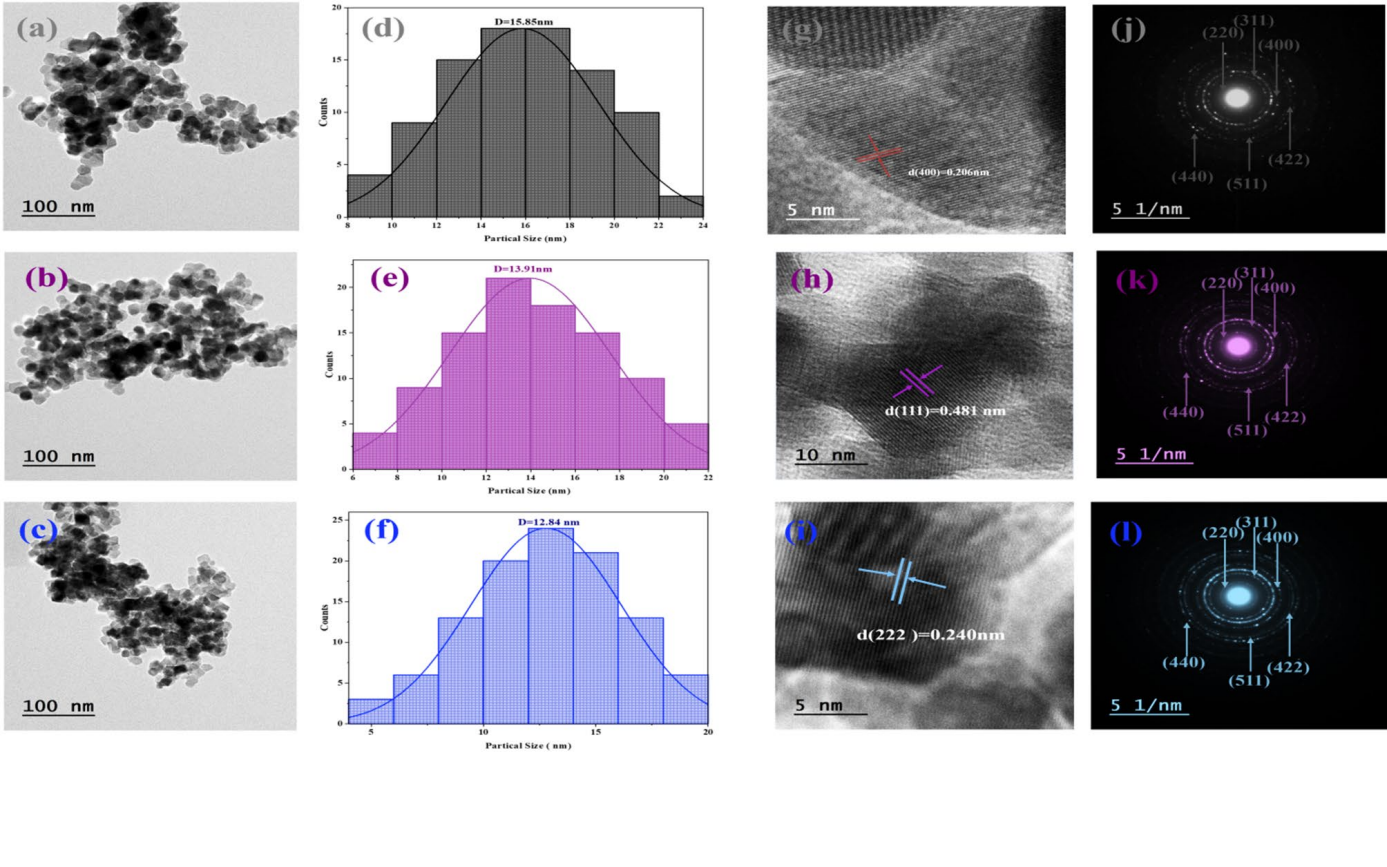
TEM analysis of Co₀.₆₋ₓZn₀.₄₋ₓNiₓAlₓFe₂O₄ nanoparticles for x = 0.00, 0.04, and 0.08: **(a–c)** TEM images showing particle morphology, **(d–f)** corresponding particle size distribution histograms, **(g–i)** high-resolution TEM (HRTEM) images revealing lattice fringes, and **(j–l)** selected area electron diffraction (SAED) patterns confirming the crystalline spinel structure.

The form and particle size of the generated nanoferrites were analyzed using transmission electron microscopy (TEM). Figure 4 (a-c) shows TEM micrographs of Co_0.6−x_Zn_0.4−x_ Ni_x_Al_x_Fe_2_O_4_ nanoparticles with x = 0.00, 0.04, and 0.08. The micrographs show a mix of spherical and cubic morphologies. It is obvious that cubic morphologies dominate as Ni^+ 2^-Al^+ 3^ co-doping increases in the doped samples, indicating the good quality of the nanocrystals. Suo et al.^43^ revealed similar cubic and spherical morphologies for Ni_0.2_Mg_0.2_Co_0.6_Fe_2−x_Al_x_O_4_ (x = 0, and 0.025) ferrites. Figure 4)d–f) depicts particle size distribution histograms created with ImageJ and Origin Lab software^44^. The TEM histograms show that the average particle size (D_TEM_) decreases from 15.85 nm for x = 0.00 to 12.84 nm for x = 0.08. This reduction is attributed to Ni²⁺–Al³⁺ co-doping, which inhibits grain growth. The smaller ionic radii and stronger local fields of the dopant ions introduce lattice strain, limiting nucleus growth and crystallite coalescence. A similar decrease in crystallite size with increasing Al³⁺ content was reported by Madhu et al.^45^ for Ni_0.3_Zn_0.5_Co_0.2_Fe_2−x_Al_x_O_4_ ferrites, where the size reduced from 36.07 to 26.57 nm as x increased to 0.20. The particle size trend obtained from TEM agrees well with the crystallite size estimated from XRD (Table 1), confirming the consistency of both characterization techniques. High-resolution TEM (HRTEM) images (Fig. 4(g–i)) show clear lattice fringes, indicating the polycrystalline nature of the nanoparticles. The measured interplanar spacing corresponds to the (400) plane of the spinel ferrite structure, consistent with XRD results. The selected area electron diffraction (SAED) patterns (Fig. 4(j–l)) exhibit concentric rings indexed to the (220), (311), (400), (422), (511), and (440) planes, confirming the cubic spinel phase. Moreover, the close agreement between lattice parameters obtained from SAED and XRD (Table 1) further validates the structural analysis.

#### SEM and EDX

**Fig. 5:**
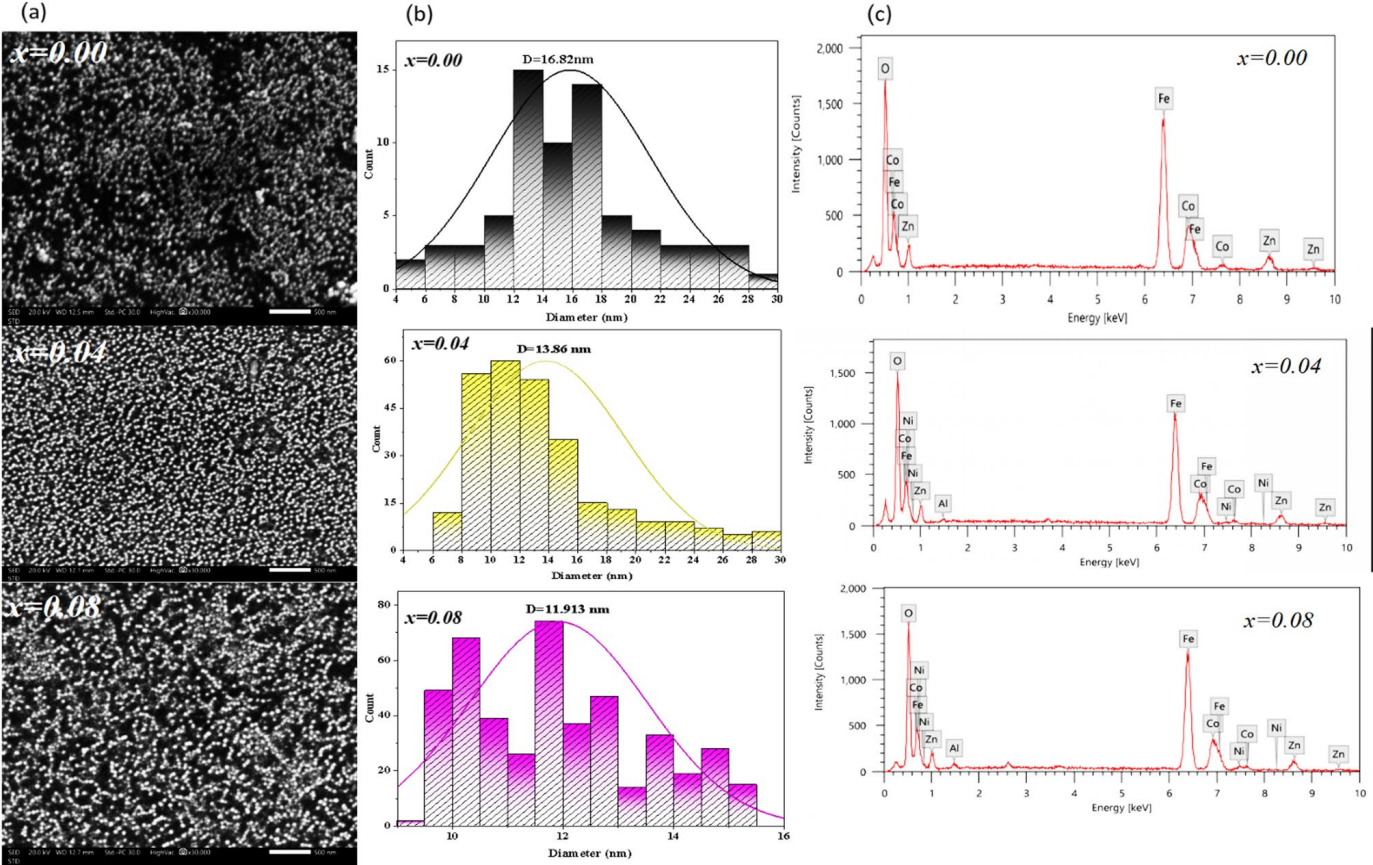
SEM analysis of Co_0.6−x_Zn_0.4−x_ Ni_x_Al_x_Fe_2_O_4_ nanoparticles for x = 0.00, 0.04, and 0.08: **(a)** SEM images showing particle morphology and aggregation, **(b)** corresponding particle size distribution histograms, and **(c)** energy-dispersive X-ray (EDX) spectra confirming elemental composition and stoichiometry.

Scanning electron microscopy (SEM) was utilized to analyze the morphology and microstructure of the generated ferrites. Figure 5(a) depicts SEM micrographs for Co_0.6−x_Zn_0.4−x_ Ni_x_Al_x_Fe_2_O_4_ with x = 0.00, 0.04, and 0.08. The uniform size of the nanoparticles indicates that the synthesis process of co-precipitation was successful. A certain amount of agglomeration is nevertheless facilitated by the magnetic attraction forces between the ferrite particles^46^. This particle aggregation in Co_0.6−x_Zn_0.4−x_ Ni_x_Al_x_Fe_2_O_4_ nanoferrites aligns with previous findings by Prabhakaran et al.^47^ for CoFe_2_O_4_ nanoparticles. The particle distribution was analyzed using ImageJ and Origin Lab software. The results show a decrease in size from 16.82 to 11.913 nm as x increases from 0.00 to 0.08, as shown in Fig. 5(b). This observation aligns with the growth trend of crystallite size predicted by the Scherrer model.

The composition, homogeneity, and elemental distribution of the synthesized nanoparticles were examined using EDX analysis. This analysis provides information about the chemical composition of Co_0.6−x_Zn_0.4−x_ Ni_x_Al_x_Fe_2_O_4_ nanoparticles (x = 0.00, 0.04, and 0.08). The EDX spectra in Fig. 5(c) confirm the presence of Co, Zn, Fe, Ni, Al, and O without any detectable impurity peaks, indicating the formation of pure ferrite samples after calcination. The elemental atomic percentages are listed in Table 2 and are close to the expected stoichiometric ratios. A slight variation in Fe content is observed with co-doping, while the atomic percentages of Co²⁺ and Zn²⁺ decrease with increasing Ni²⁺ and Al³⁺ concentration, which agrees with the nominal composition. These results confirm the successful synthesis and good homogeneity of the Co_0.6−x_Zn_0.4−x_ Ni_x_Al_x_Fe_2_O_4_ nanoparticles.

**Table 2:** Elemental atomic percentages of Co_0.6−x_Zn_0.4−x_ Ni_x_Al_x_Fe_2_O_4_ nanoparticles for x = 0.00, 0.04, and 0.08, obtained from energy-dispersive X-ray (EDX) analysis, confirming the stoichiometry and composition of the synthesized samples.

Element	Atomic %		
	x = 0.00	x = 0.04	x = 0.08
O	53.91 ± 0.55	53.25 ± 0.47	53.81 ± 0.5
Fe	30.65 ± 0.32	31.07 ± 0.28	31.48 ± 0.30
Co	10.02 ± 0.21	9.04 ± 0.18	8.26 ± 0.18
Zn	5.43 ± 0.20	5.29 ± 0.17	4.33 ± 0.17
Ni	0.0	0.77 ± 0.07	1.36 ± 0.09
Al	0.0	0.58 ± 0.07	0.77 ± 0.09

### Optical properties

#### UV-vis spectroscopy

**Fig. 6:**
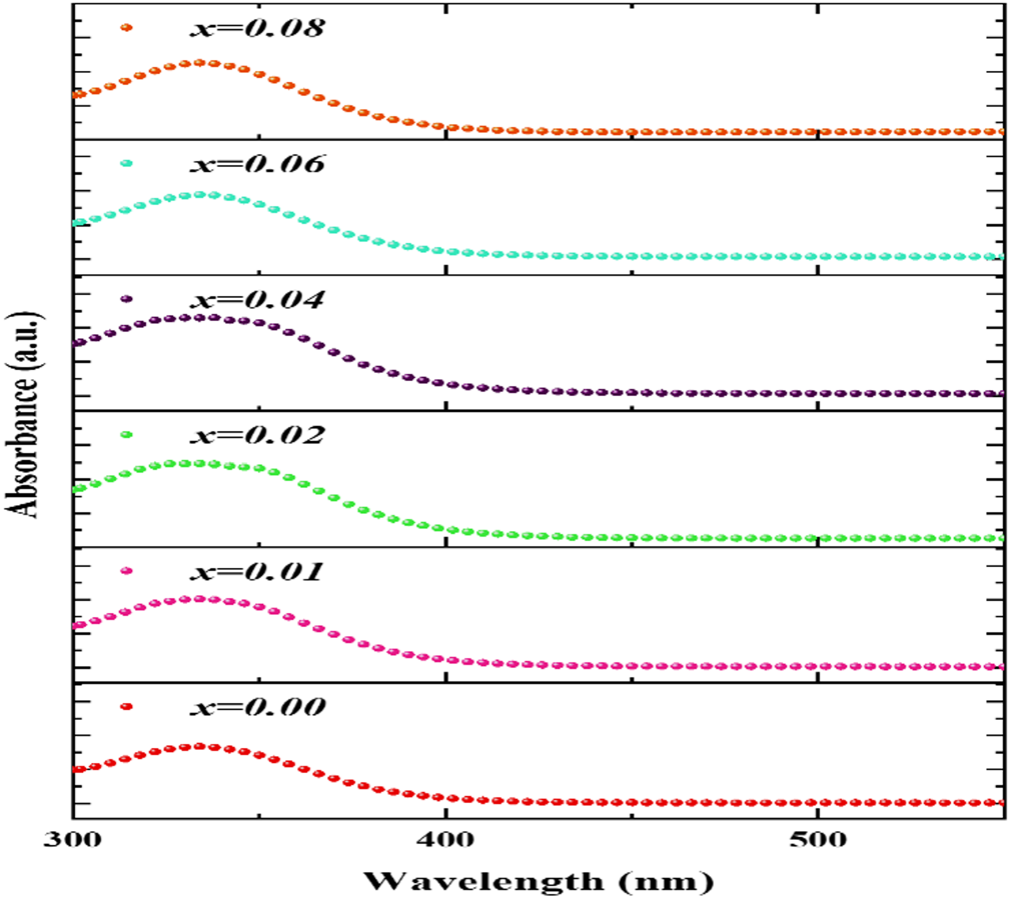
UV–visible absorbance spectra of Co₀.₆₋ₓZn₀.₄₋ₓNiₓAlₓFe₂O₄ nanoparticles, showing the systematic blue shift in absorption with increasing Ni²⁺–Al³⁺ co-doping concentration (x).

Figure 6 displays room-temperature UV-visible absorption spectra, with wavelengths ranging from 300 to 550 nm, for Co_0.6−x_Zn_0.4−x_Ni_x_Al_x_Fe_2_O_4_ ferrites with x = 0.00, 0.01, 0.02, 0.04, 0.06, and 0.08. The optical characteristics of the produced nanoparticles, including their bandgap energy (E_g_), Urbach energy (E_U_), electron-photon interaction strength$\:{\:(\mathrm{E}}_{\mathrm{e}-\mathrm{p}\mathrm{h}}),\mathrm{v}\mathrm{a}\mathrm{l}\mathrm{e}\mathrm{n}\mathrm{c}\mathrm{e}\:\mathrm{b}\mathrm{a}\mathrm{n}\mathrm{d}\:\left({\mathrm{E}}_{VB}\right),\:\mathrm{a}\mathrm{n}\mathrm{d}\:\mathrm{c}\mathrm{o}\mathrm{n}\mathrm{d}\mathrm{u}\mathrm{c}\mathrm{t}\mathrm{i}\mathrm{o}\mathrm{n}\:\mathrm{b}\mathrm{a}\mathrm{n}\mathrm{d}\:\left({\mathrm{E}}_{CB}\right)\mathrm{e}\mathrm{d}\mathrm{g}\mathrm{e}\mathrm{s},$ were examined as a function of Ni^+ 2^-Al^+ 3^ co-doping content. Each sample displays a primary absorption peak between 335 and 337 nm, as shown from Fig. 6. Intrinsic band gap absorption, which is connected to electron transfers from the valence band to the conduction band, is what causes this absorption peak^48^. Previous studies have shown similar UV-vis spectra of ferrite nanoparticles, namely ZnFe_2_O_4_, CoFe_2_O_4_, and Zn_0.5_Mg_0.5_Fe_2_O_4_^49–51^. When the peak approaches the UV zone as a result of a rise in Ni^2+^ and Al^3+^ concentration, a blue shift takes place. It is crucial to keep in mind that the doping procedure may alter the size and structure of the ferrite nanoparticles. Because of the quantum confinement effect, smaller nanoparticles have greater band gap energies^52,53^.

**Fig. 7:**
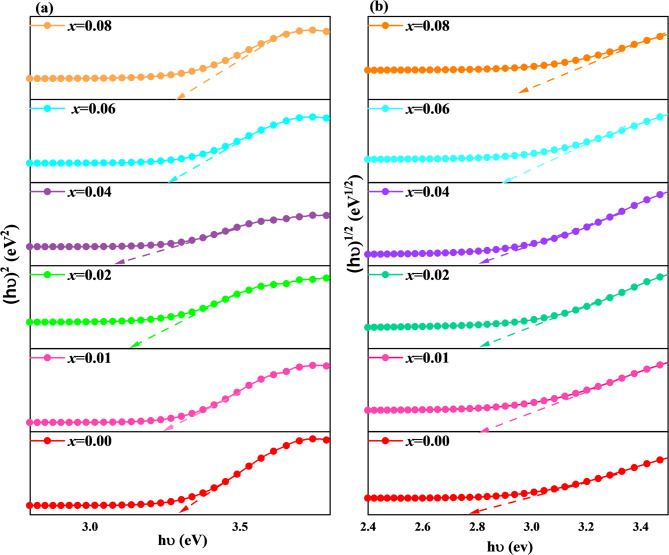
Variation of **(a)** direct and **(b)** indirect bandgap energies of Co_0.6−x_Zn_0.4−x_Ni_x_Al_x_Fe_2_O_4_ nanoparticles as a function of Ni²⁺–Al³⁺ co-doping concentration (x).

**Fig. 8:**
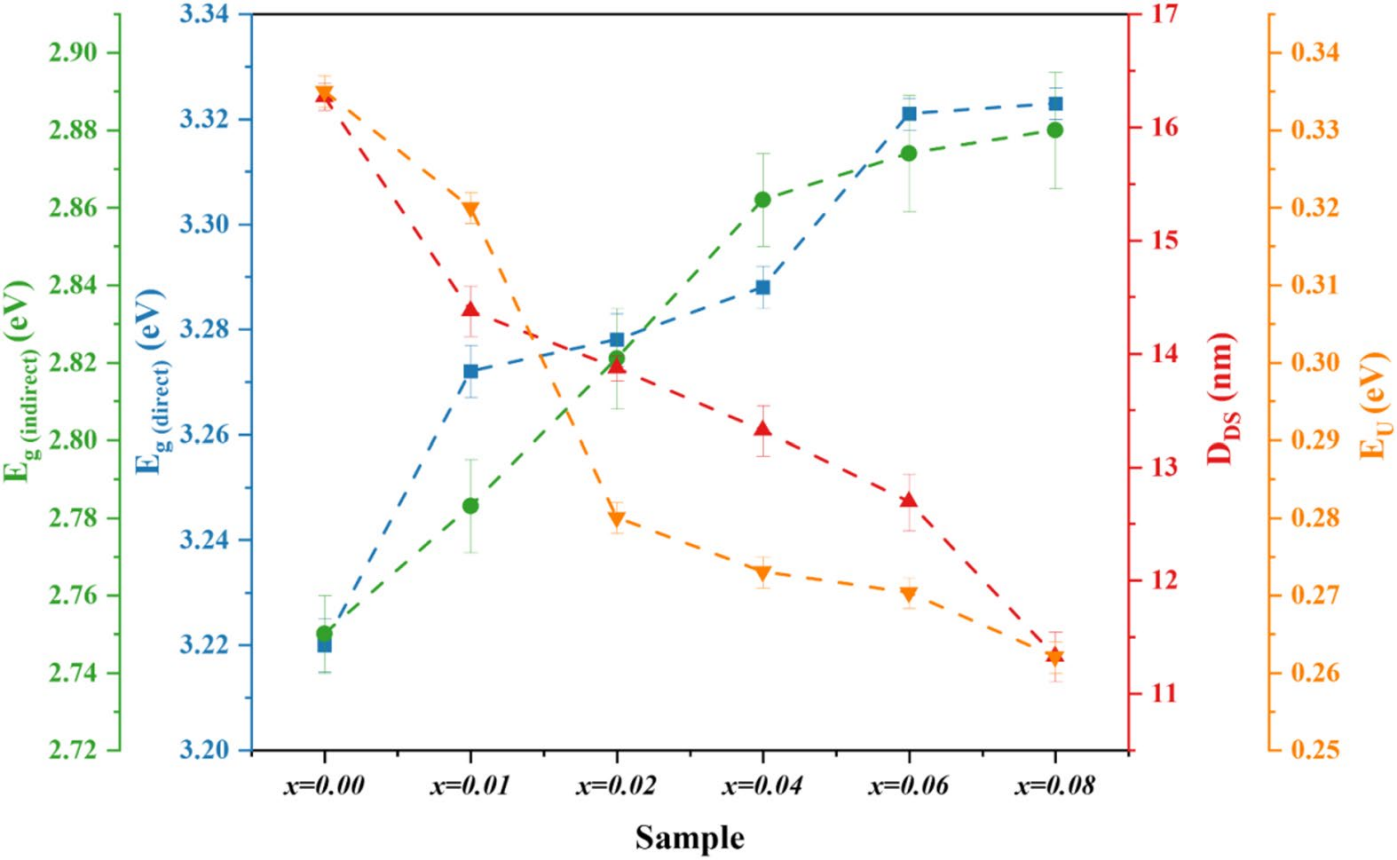
Variations of direct bandgap energy, indirect band-gap energy, E_U_, and D_DS_ for Co_0.6−x_Zn_0.4−x_Ni_x_Al_x_Fe_2_O_4_ nanoparticles.

Using Tauc’s equation^54^, the optical band-gap energy (E_g_) of the produced nanoparticles was calculated as follows:

7 $$\:{\:\:\left(\alpha\:h\upsilon\:\right)}^{n}=B\left(h\upsilon\:-{E}_{g}\right),$$

where the photon energy, absorption coefficient, and transition probability dependence constant are represented by the symbols$\:\:h\upsilon\:,\:B,$ and $\:\alpha\:$, respectively. Additionally, several kinds of electronic transitions are linked to the exponent n. A direct transition occurs when *n* = 2, while an indirectly transition occurs when *n* = 0.5. The absorption coefficient is computed using the following relation^55^:

8 $$\:{\upalpha\:}=\frac{2.303\:\mathrm{A}}{\mathrm{d}}$$

where the absorbance is represented by A, and the length of the light path by (= 1 cm). The band-gap energy is determined by extending the linear segment to the energy axis from the (ℎ)^2^ and (ℎ)^1/2^ versus ℎ, as shown in Fig. 7 (a) and (b). As shown in Fig. 8, the direct and indirect bandgap energies increase from 3.220 to 3.323 eV and from 2.75 to 2.88 eV, respectively, as the co-doping level rises from 0.00 to 0.08. Figure 8 also reveals an inverse relationship between bandgap energy and crystallite size. These results agree with the XRD and SEM analyses and can be explained by the quantum confinement effect^52,53^. A similar trend was reported when Ba doping in CoFe_2_O_4_ reduced particle size and increased the bandgap energy^56^.

**Fig. 9:**
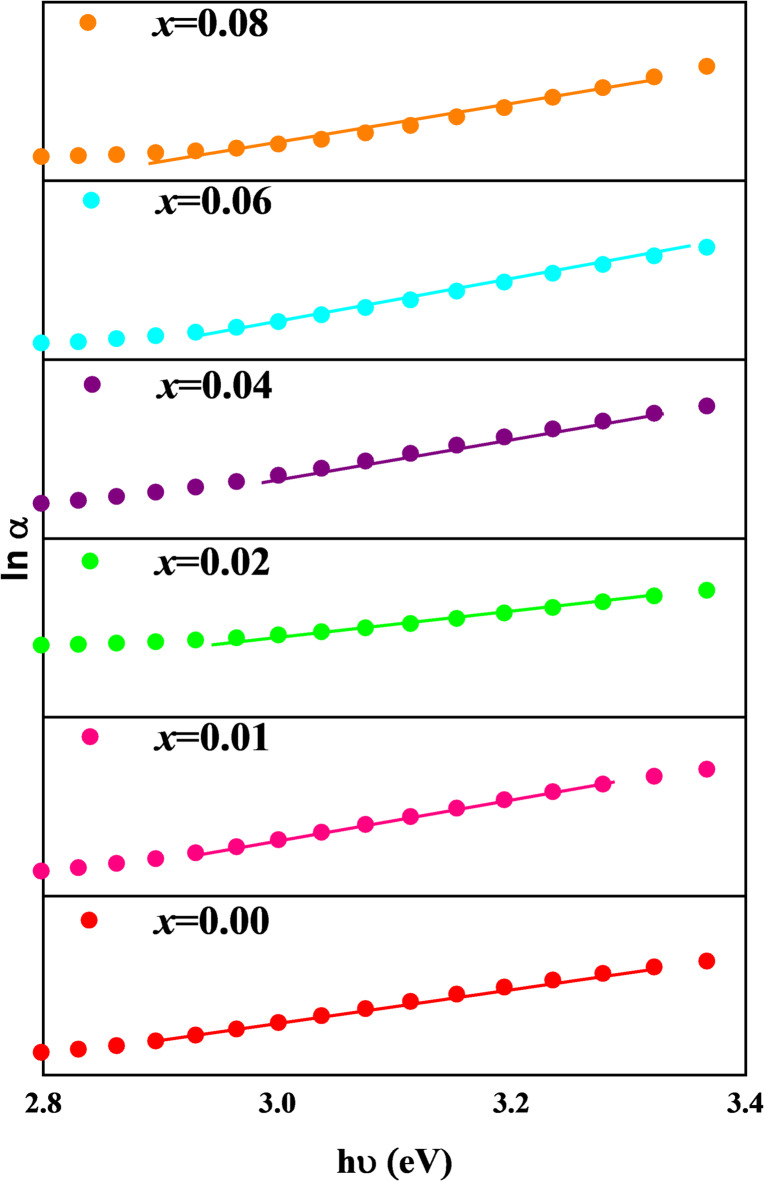
Urbach energy plot for Co_0.6−x_Zn_0.4−x_Ni_x_Al_x_Fe_2_O_4_ nanoparticles.

The disorder and band tail width of the localized states inside the energy gap are measured by the Urbach energy (E_U_). This formula^57^ may be used to determine the E_U_:

9 $$\:\mathrm{ln}\left({\upalpha\:}\right)=\:\mathrm{ln}{({\upalpha\:}}_{0})+\:\frac{\mathrm{h}{\upupsilon\:}}{{\mathrm{E}}_{\mathrm{U}}}$$

where α_0_ is a constant. As shown in Fig. 9, the E_U_ values were computed by calculating the reciprocal of the slope of the linear plots of ln versus ℎ. In Fig. 8, the estimated E_u_ values are compared with direct band-gap energy, indirect band-gap energy, and D_DS_. It is evident that E_U_ falls from 0.335 to 0.262 eV when x rises from 0.00 to 0.08. This implies that the band structure is less disordered, which explains why bandgap energies increase with increasing concentrations of Ni^2+^ and Al^3+^. Furthermore, Fig. 8 shows a negative association between the E_U_ and both direct and indirect band gap energies.

A physical characteristic of the bandgap that describes the thickening of the absorption edge due to electron-phonon interactions is the steepness parameter (σ_st_)^58^. It is measured using the following formula^59^:

10 $$\:{{\upsigma\:}}_{\mathrm{s}\mathrm{t}}=\frac{{\mathrm{k}}_{\mathrm{B}\:}\mathrm{T}}{{\mathrm{E}}_{\mathrm{U}}}$$

where T is the ambient temperature, and k_B_ is the Boltzmann constant. Additionally, the electron-photon interaction strength $\:{\mathrm{E}}_{\mathrm{e}-\mathrm{p}\mathrm{h}}$ may be calculated using the following relation, which is inversely proportional to the steepness parameter^60^:

11 $$\:{\mathrm{E}}_{\mathrm{e}-\mathrm{p}\mathrm{h}}=\frac{2}{3{{\upsigma\:}}_{\mathrm{s}\mathrm{t}}}$$

The $\:{{\upsigma\:}}_{\mathrm{s}\mathrm{t}}$ and $\:{\mathrm{E}}_{\mathrm{e}-\mathrm{p}\mathrm{h}}$ values for the Co_0.6−x_Zn_0.4−x_Ni_x_Al_x_Fe_2_O_4_ samples are listed in Table 3. As x increases from 0.00 to 0.08, $\:{{\upsigma\:}}_{\mathrm{s}\mathrm{t}}$ increases from 0.076 to 0.098 while $\:{\mathrm{E}}_{\mathrm{e}-\mathrm{p}\mathrm{h}}\:$decreases from 8.696 to 6.801. Co_0.4_Zn_0.4_Ni_0.08_Al_0.08_Fe_2_O_4_ nanoparticles had the lowest electron-phonon interaction and the highest steepness value among the generated nanoparticles. The increased steepness parameter and decreased electron-phonon interaction energy suggest that co-doping improves crystalline quality and lowers the density of certain structural flaws in the generated nanoparticles^61^.

**Fig. 10:**
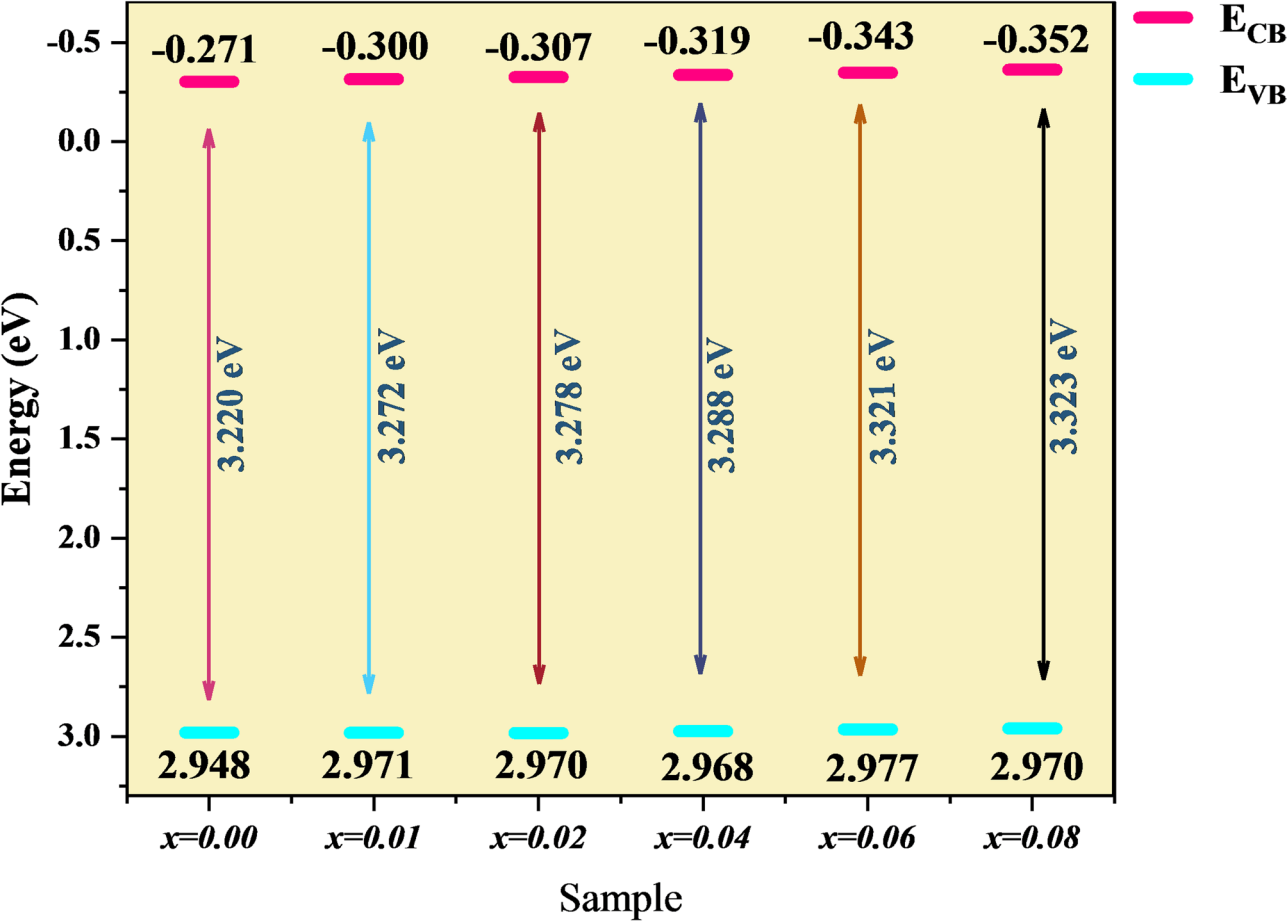
Schematic illustration of the valence band (E_VB_) and conduction band (E_CB_) for Co_0.6−x_Zn_0.4−x_Ni_x_Al_x_Fe_2_O_4_ nanoparticles, showing the effect of Ni²⁺–Al³⁺ co-doping on the band structure and electronic transitions.

The valence band (E_VB_) and conduction band (E_CB_) edges, which are seen in Fig. 10^60^, were calculated using the following equations:

12 $$\:{\mathrm{E}}_{\mathrm{C}\mathrm{B}\:}={\upchi\:}-{\mathrm{E}}^{\mathrm{c}}-{0.5\mathrm{E}}_{\mathrm{g}}$$

13 $$\:{\mathrm{E}}_{\mathrm{V}\mathrm{B}}=\:{\mathrm{E}}_{\mathrm{C}\mathrm{B}}+{\mathrm{E}}_{\mathrm{g}}$$

where E_g_ is the direct bandgap energy, and E^c^ is the energy of the free electrons on the hydrogen scale, which is equivalent to 4.5 eV. Furthermore, the following formula yields the absolute electronegativity^61^:

14 $$\:\chi\:=\:{\left[\chi\:{\left(Co\right)}^{0.6-x}\chi\:{\left(Zn\right)}^{0.4-x}\chi\:{\left(Ni\right)}^{x\:}\chi\:{\left(Al\right)}^{x\:}\chi\:{\left(Fe\right)}^{2\:}\chi\:{\left(O\right)}^{4\:}\right]}^{\frac{1}{y}}$$

where represents the entire number of atoms (7) in the formula Co_0.5_Zn_0.3_Ni_0.1_Al_0.1_Fe_2_O_4_. Additionally, the arithmetic mean of the electron affinity and initial ionization energy is used to calculate the value of χ for each atom. As a result, the corresponding χ values for Co, Zn, Ni, Al, Fe, and O are 4.27, 4.40, 4.40, 3.21, 4.03, and 7.54 eV.

The following formula was used to determine the UV resonance energy (E_0_)^62^:

15 $$\:{\mathrm{E}}_{0}={\mathrm{E}}_{\mathrm{g}}+{\mathrm{I}}_{\mathrm{H}}$$

where I_H_ is the ionization energy of the hydrogen atom. Table 3 shows that the addition of Ni and Al co-dopants to Co_0.6_Zn_0.4_Fe_2_O_4_ nanoparticles causes a little rise in E_0_ from 16.82 to 16.923. The rise in band-gap energy is the main cause of this.

**Table 3:** The optical parameters for Co_0.6−x_Zn_0.4−x_Ni_x_Al_x_Fe_2_O_4_ nanoparticles.

Parameter	Sample					
	x = 0.00	x = 0.01	x = 0.02	x = 0.04	x = 0.06	x = 0.08
σ_st_	0.076	0.0802	0.091	0.094	0.095	0.098
E_e−ph_	8.696	8.307	7.269	7.087	7.017	6.801
E_ο_	16.821	16.872	16.878	16.888	16.921	16.923

#### FTIR analysis

**Fig. 11:**
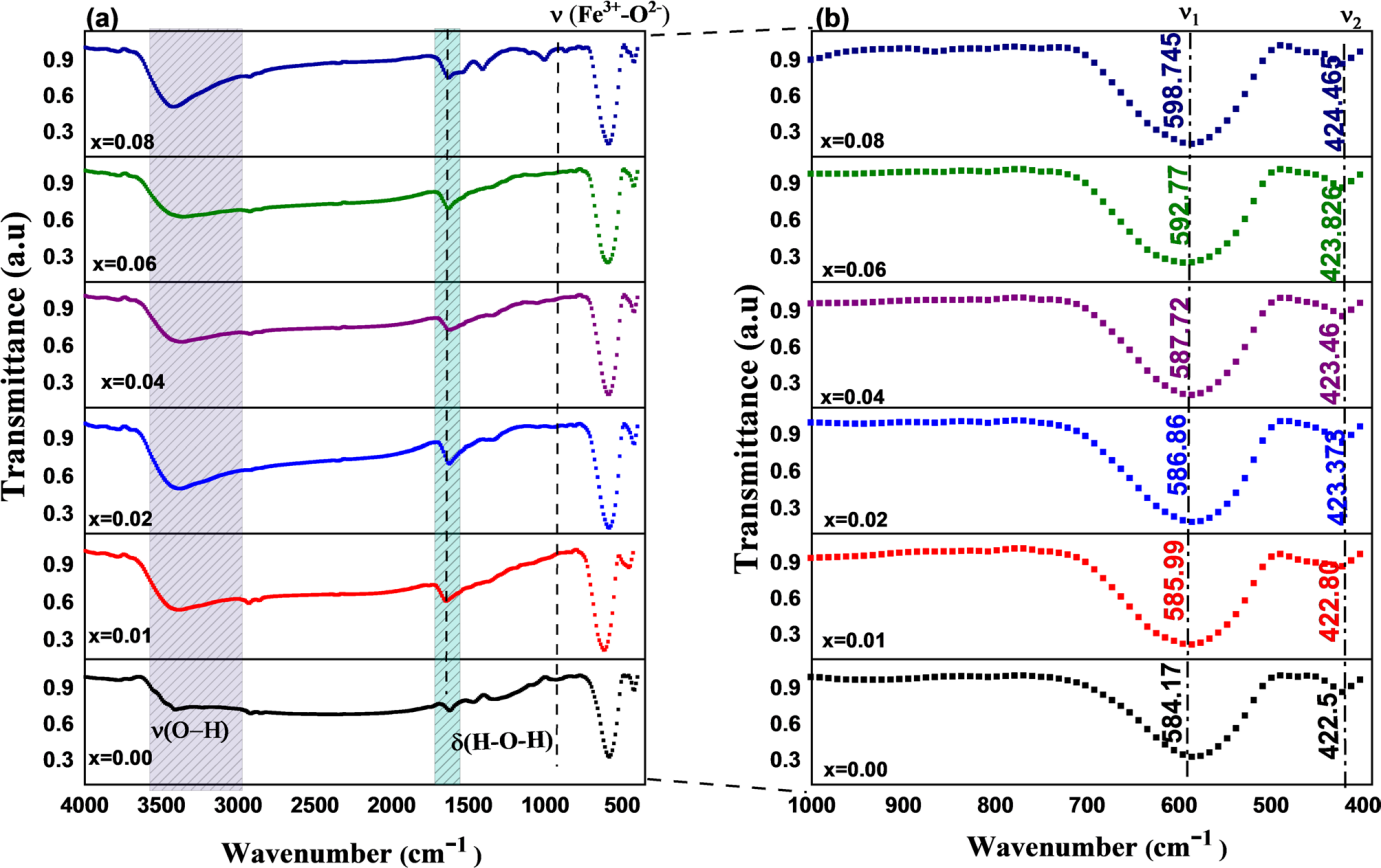
Fourier transform infrared (FTIR) spectra of Co₀.₆₋ₓZn₀.₄₋ₓNiₓAlₓFe₂O₄ nanoparticles: (**a**) full spectral range showing characteristic metal–oxygen vibrations, and (**b**) zoomed-in view of the fingerprint region highlighting spinel-specific bands.

Figure 11(a) displays the room temperature FTIR spectra of Co₀.₆₋ₓZn₀.₄₋ₓNiₓAlₓFe₂O₄ ferrites (x = 0.00, 0.01, 0.02, 0.04, 0.06, and 0.08), encompassing the wavenumber range of 350–4000 cm⁻¹. Infrared (IR) radiation analysis of atomic and molecular vibrations provides valuable information on local chemical bonding and short-range order in ferrite structures. The stretching υ(O–H) and bending δ(H–O–H) vibrations of adsorbed water molecules linked to surface hydroxyl groups are responsible for the broad absorption band around ~ 3415 cm⁻¹ and the weak one near ~ 1640 cm⁻¹^63^. This indicates that the samples are hygroscopic, meaning they readily absorb moisture from the air when exposed; the presence of ambient CO_2_ is attributed to a narrow band around 2360 cm⁻¹^64^. The spectral characteristics highlight low-oxygen regions of the surface, increasing the number of active sites for molecule adsorption. The distribution of cations between the tetrahedral (A) and octahedral (B) sites has a major effect on the FTIR spectra of spinel ferrites^65^. There are two main absorption bands in the fingerprint area (350–600 cm⁻¹), as shown in Fig. 11(b). The higher frequency band (υ₁ = 584.17–598.75 cm⁻¹) is caused by stretching vibrations of metal–oxygen bonds at tetrahedral sites, whereas the lower frequency band (υ₂ ≈ 422.50–424.47 cm⁻¹) is caused by octahedral sites^66^. The presence of these two bands confirms the formation of a single-phase cubic spinel structure. When Ni^2+^-Al^3+^ ions are gradually replaced for Co²⁺ and Zn²⁺, υ_1_ and υ_2_ show a systematic blue shift. Notably, υ_1_ increases from 584.17 cm⁻¹ (x = 0.00) to 598.75 cm⁻¹ (x = 0.08), while υ_2_ shifts from 422.50 cm⁻¹ to 424.47 cm⁻¹. This shift, which is associated with the shorter ionic radius and lighter mass of Ni^2+^-Al^3^ compared to Co²⁺/Zn²⁺, shortens the Fe³⁺–O²⁻ bond length and enhances bond stiffness^65^. The average vibrational frequency rises from 503.34 cm⁻¹ to 506.06 cm⁻¹ in line with the connection between vibrational frequency and cation mass, bond length, and binding strength. A similar blue shift of the tetrahedral and octahedral vibrational bands upon Al³⁺ substitution has also been reported in Ni–Cu ferrites^67^, indicating that the observed shift is due to mass reduction and lattice stiffening effects.

**Fig. 12:**
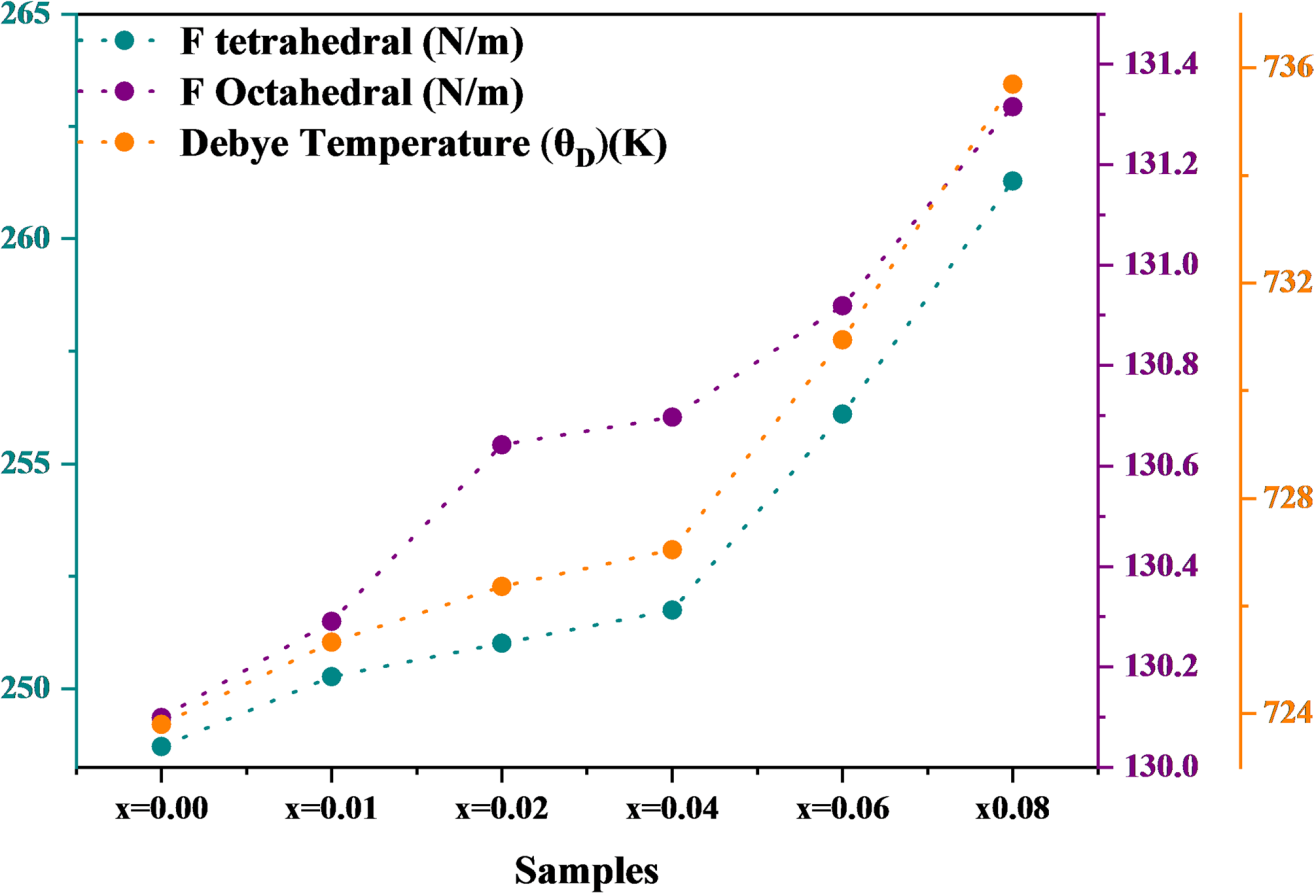
variation of octahedral (F_Oh_) and tetrahedral (F_Td_) force constants and Debye temperature (θ_D_) of Co₀.₆₋ₓZn₀.₄₋ₓNiₓAlₓFe₂O₄ nanoparticles as a function of Ni²⁺–Al³⁺ co-doping concentration (x), illustrating the influence of doping on lattice dynamics and bonding strength.

The following formula was used to determine the force constant (F)^68^:

16 $$\:F\hspace{0.17em}=\hspace{0.17em}42c22$$

where µ is the reduced mass of Fe^3+^ and O^2−^ ions (µ = 2.061 1026 kg), c is the speed of light in vacuum, and υ is the sublattice frequency^55^ The force constants, denoted as F_Oh_ for the octahedral site and F_Td_ for the tetrahedral site, are shown in Fig. 12. The rise in both FTIR-derived force constants with Ni^2+^-Al^3+^ co-doped ferrites (F_Td_: 248.72 to 261.284 N m⁻¹; F_Oh_: 130.10 to 131.3155 N m⁻¹ for x = 0.00 to 0.08) reflects lattice stiffening at both the A- and B-sublattices. This rise in force constants indicates stronger and more rigid metal–oxygen bonds in both sublattices. These changes most likely result from the redistribution of cations inside the lattice^69^. The Debye temperature (θ_D_) is a crucial metric in solid-state physics because it clearly shows the lattice vibrational characteristics of crystalline materials^69^. The following formula was used to determine the Debye temperature of the produced nanoparticles^57^:

17 $$\:{\:\:\:\:\:{\uptheta\:}}_{\mathrm{D}}=\mathrm{h}\mathrm{c}{v}_{\mathrm{a}\mathrm{v}}/{\mathrm{k}}_{\mathrm{B}}$$

where $\:{v}_{\mathrm{a}\mathrm{v}}$ (m^− 1^) is the average wavenumber of vibrational bands, $\:{\mathrm{k}}_{\mathrm{B}}$ is the Boltzmann constant ($\:{\mathrm{k}}_{\mathrm{B}}$ = 1.3806 × 10^− 23^ J.K^− 1^), and $\:\mathrm{h}$ represents the Planck’s constant ($\:\mathrm{h}$ = 6.626 × 10^− 34^ J.s). The computed Debye temperature values (θ_D_) for the synthesized samples are shown in Fig. 12.

As the co-dopant concentration increases from x = 0.00 to x = 0.08, the Debye temperature (θ_D_) of the synthesized samples rises from 723.796 K to 735.695 K. The smaller ionic radii and lower atomic masses of Ni²⁺–Al³⁺ compared with Co²⁺/Zn²⁺ shorten the M–O bond length, strengthen bond stiffness, and suppress lattice vibrations. Consequently, θ_D_ increases. This trend agrees with the higher force constants (F_Td_, F_Oh_) obtained from FTIR analysis^70^, indicating that Ni²⁺–Al³⁺ co-doping enhances interatomic bonding and stiffens the spinel lattice. Similar behavior has been reported for Al³⁺-substituted Ni–Zn ferrites, where stronger bonding resulted in higher θ_D_ and elastic modulus^63^. In another study on Ni–Cd micro-ferrites, the Debye temperature increased from 450.58 K to 500.50 K as the Ni and Cd content rose from 0.0 to 0.6^71^. Overall, these results suggest that Ni²⁺–Al³⁺ co-doping enhances lattice stiffness and thermodynamic stability, thereby improving the structural robustness of the Co–Zn spinel lattice.

#### Raman analysis

**Fig. 13:**
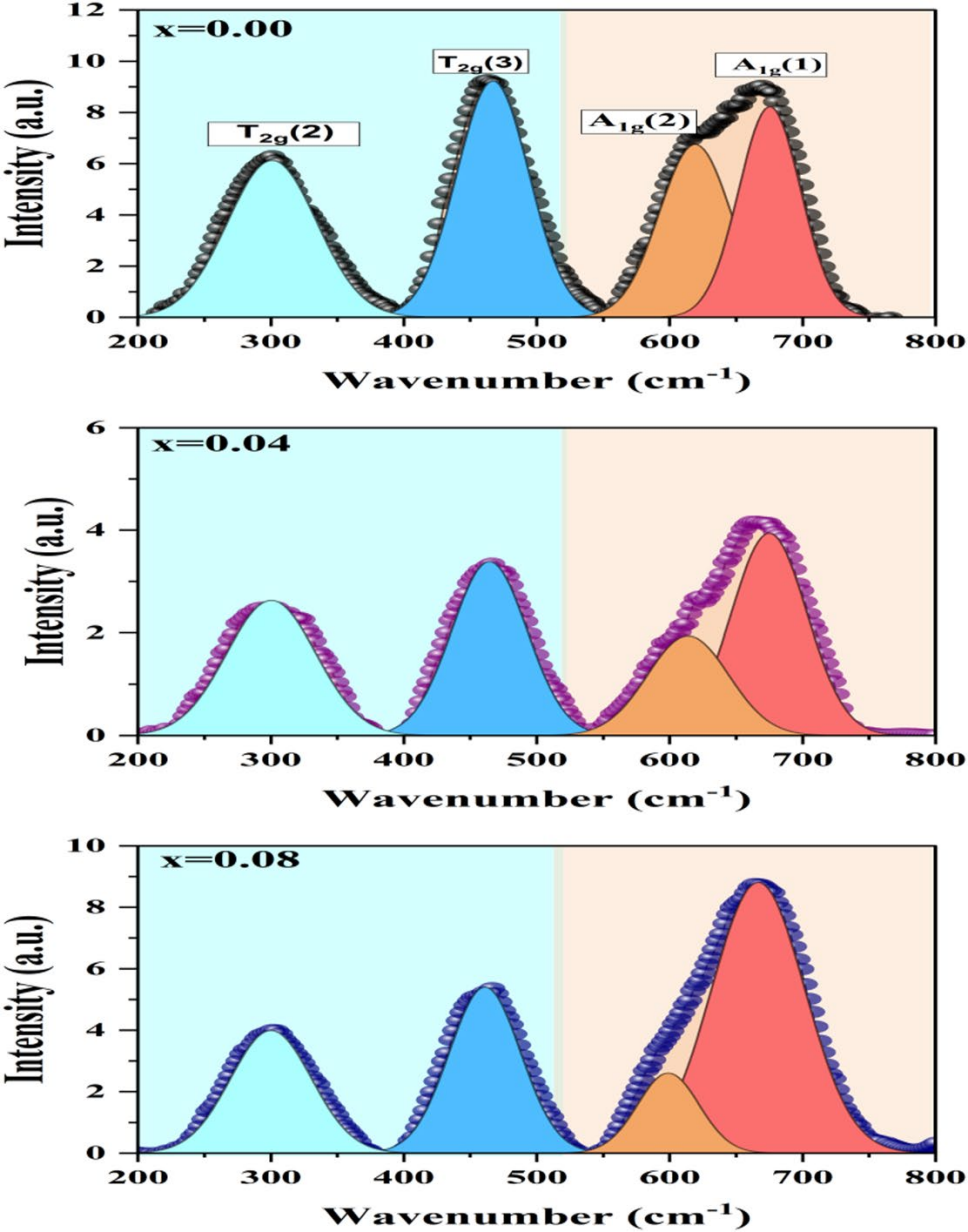
Deconvoluted Raman spectra of Co₀.₆₋ₓZn₀.₄₋ₓNiₓAlₓFe₂O₄ nanoparticles (x = 0.00, 0.04, 0.08), showing spinel vibrational modes and shifts caused by Ni²⁺–Al³⁺ co-doping.

Raman spectroscopy is used to identify the vibrational modes of the synthesized spinel nanoferrites. Figure 13 displays the deconvolution of the room temperature Raman spectra of the chosen samples (x = 0.00, 0.04, and 0.08) in the 200–800 cm^− 1^ region. The peak position values are similar to those seen in the literature^72–74^. The Fd$\:\stackrel{-}{3}$m space group of the cobalt-zinc ferrite nanoparticles allows them to generate 39 vibrational modes. As seen in Fig. 13, spinel ferrites were linked to four of the 39 Raman active modes, 2A_1g_ and 2T_2g_. The formation of a cubic phase is indicated by the appearance of Raman-active peaks in the treated samples. The movement of oxygen and metal ions at the A-site and B-site produces the spinel structure’s active modes. The mobility between the oxygen and the tetrahedral site is shown by a Raman spectra frequency greater than 600 cm^− 1^. The octahedral site and oxygen are moving if the frequency is less than 600 cm^− 1^. A_1g_ is associated with the symmetric stretching of the oxygen anion across the Fe/M-O bond at the tetrahedral site, whereas T_2g_ is associated with the vibrations of the octahedral site^73^. The presence of different cations is indicated by the broad-shouldered peak that correlates to the A_1g_ mode in the Al- and Ni-co-doped Co-Zn nanoferrites. These cations have the same symmetry and are all in the same place within the tetrahedral site^75^. The stretching vibrations of the M-O bond in the tetrahedral coordination, represented by the A_1g_(2) mode, are indicated by the peak at about 625 cm^− 1^.

The asymmetric stretching vibration of the M-O bond is shown by the peak at about 475 cm^− 1^, which is consistent with the T_2g_(1) mode. It implies that within the spinel lattice, the metal and oxygen ions are travelling in different directions^76^. The antisymmetric bending vibrations of oxygen ions that correspond to the metal cations in the tetrahedral unit are represented by the peak at around 330 cm^− 1^, which is known as the T_2g_(2) mode^76^. The slight red shift in the location of this peak is seen in Fig. 13. It demonstrates that the change in phase content is primarily responsible for the variance in peak intensity and location^77–79^.

### XPS analysis

**Fig. 14:**
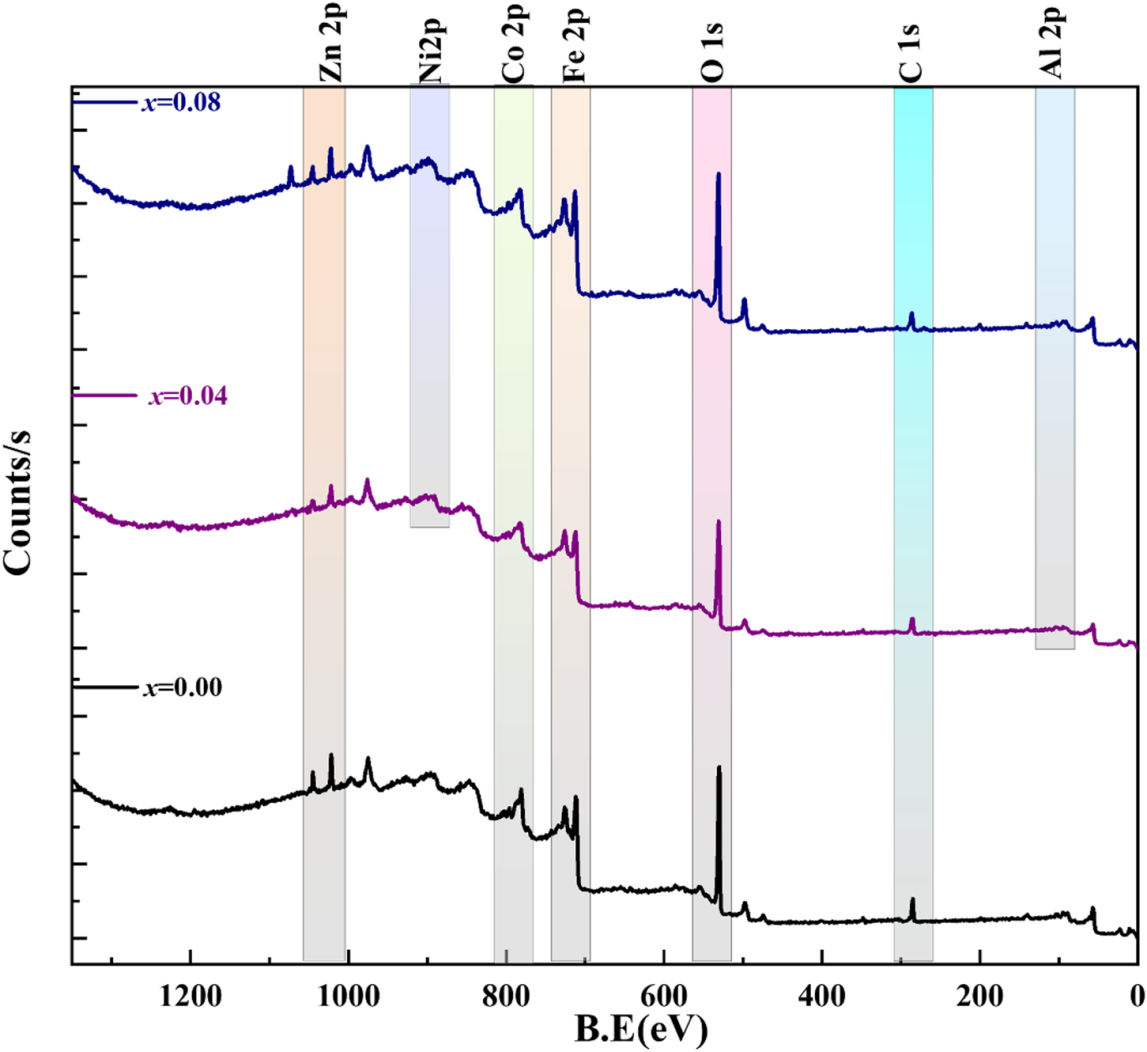
XPS Spectra of Co_0.6−x_Zn_0.4−x_Ni_x_Al_x_Fe_2_O_4_ nanoparticles (*x* = 0.00, 0.04, and 0.08).

X-ray photoelectron spectroscopy (XPS) is used to determine the elemental valence states and cation distribution of the spinel structure’s tetrahedral and octahedral sites. Figure 14 displays the whole Co_0.6−x_Zn_0.4−x_Ni_x_Al_x_Fe_2_O_4_ XPS survey spectrum with x = 0.00, 0.04, and 0.08. The distinctive peaks that verify the presence and valence states of these crucial elements in the pure sample (x = 0.00) are Co, Zn, Fe, O, and C. New peaks associated with the Ni and Al elements emerge as they are co-doped. The C 1 s peak at 285 eV will be caused by adventitious carbon contamination on the sample surface, which is linked to C–C or C–H bonds^80^. The spontaneous C 1 s peak at 285 eV has been used as a reference to calibrate the binding energies of all discovered elements. Additional photoemission peaks that correspond to the binding energies of Ni 2p and Al 2p electrons in the XPS spectra of Ni^2+^-Al^3+^ co-doped materials clearly demonstrate that Ni and Al have been successfully integrated into the host structure. The absence of spectral features from elements other than Co, Zn, Fe, Ni, Al, and O in the XRD and Raman data indicates the high material purity.

**Fig. 15:**
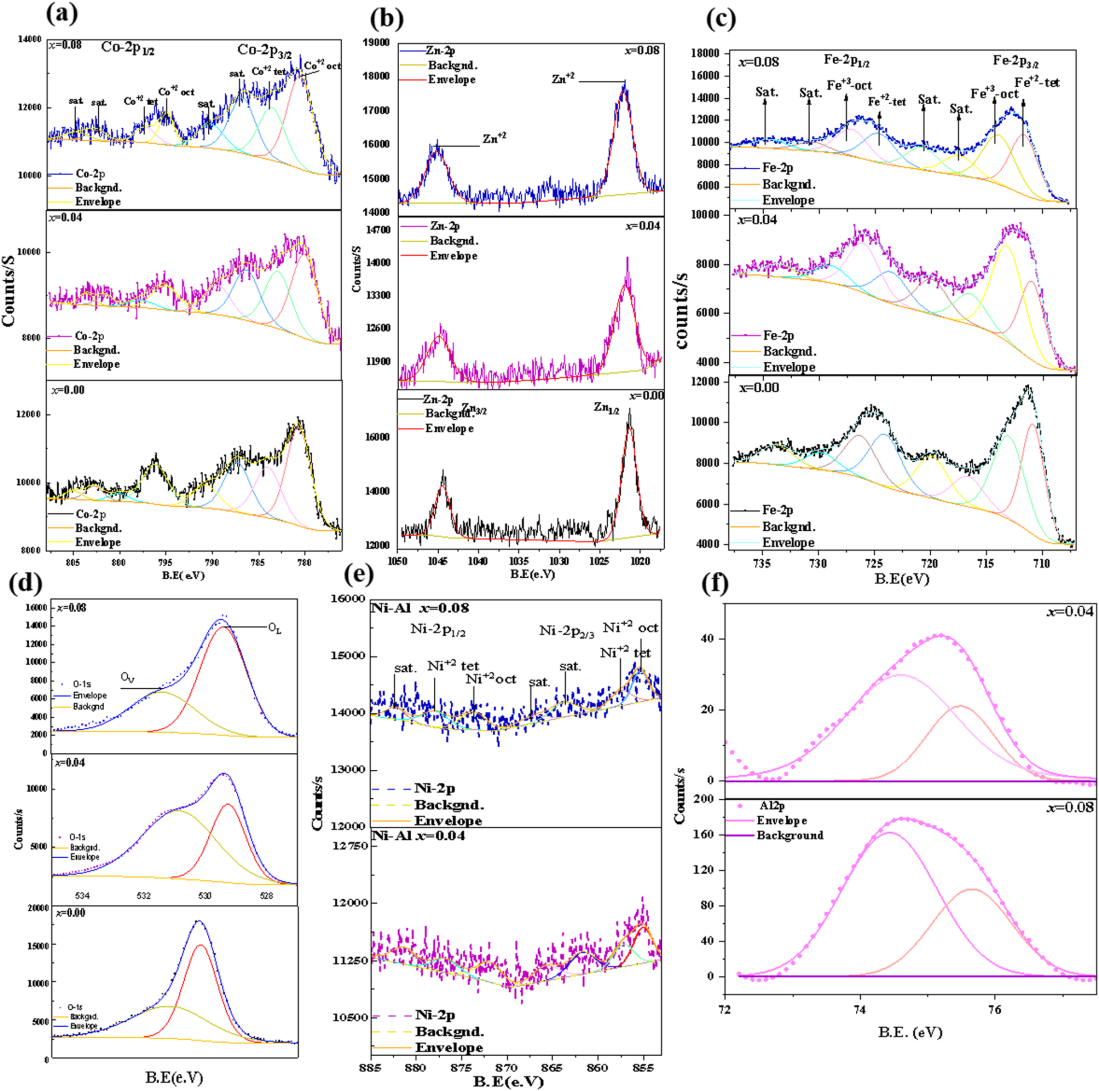
Deconvoluted XPS spectra of **(a)** Co-2p, **(b)** Zn-2p, **(c)** Fe-2p, **(d)** O-1s, **(e)** Ni-2p, and **(f)** Al-2p, of Co_0.6−x_Zn_0.4−x_Ni_x_Al_x_Fe_2_O_4_ nanoparticles (*x* = 0.00, 0.04, and 0.08).

As seen in Fig. 15(a–f), the high-resolution XPS (HRXPS) spectra for individual elements are deconvoluted using Fit-yk software to evaluate the core energy levels. Each element’s unique XPS spectra indicate that the 2p energy level splits into two states, 2p_3/2_ and 2p_1/2_, due to spin-orbit interaction^81^. Figure 15(a) displays the HRXPS analysis of the Co 2p peaks. Deconvolution of the 2p peak areas reveals two distinct bonding corresponding to the two types of lattice sites, octahedral and tetrahedral. The binding energies of Co 2p_3/2_ are 780 eV and 783 eV. Co^2+^ generates the peak at 780 eV in octahedral coordination with oxygen but at 783 eV in tetrahedral coordination. Additionally, Co 2p_1/2_ is connected with peaks at octahedral and tetrahedral sites at around 795 eV and 798 eV, respectively. Furthermore, satellite peaks with larger binding energies in the 2p3/2 and 2p1/2 regions of the XPS spectra confirm the + 2 oxidation state of Co^82,83^. Also, the integral area of the peak at 781 eV is significantly larger than that of the peak at 783 eV, indicating that octahedral sites are mostly occupied by Co^2+^. Thus, the octahedral to tetrahedral peak area ratio rises as the concentration of co-doping increases, indicating that Co ions are moving from the tetrahedral to the octahedral location. The results of the Raman study are consistent with this.

As shown in Fig. 15(b), the high-resolution XPS spectra of Zn 2p for the selected samples seem to exhibit two prominent peaks at around 1021 eV and 1044 eV, which correspond to Zn 2p_3/2_ and Zn 2p_1/2_, respectively. These peaks show that zinc is present in the 2 + oxidation state^84^. The Fe 2p_1/2_ spectrum displays two peaks at about 724 eV and 727 eV, while the Fe 2p_3/2_ spectrum deconvolutes into two distinct peaks with binding energies of approximately 710 eV and 713 eV, according to Fig. 15(c). The presence of two distinct Fe ion bonds indicates two types of lattice sites for Fe ion occupancy: tetrahedral sites, which are associated with higher binding energy, and octahedral sites, which are associated with lower binding energy^85^. Furthermore, the small satellite peaks at 719 eV and 734 eV are caused by electronic transitions caused by charge transfer between Fe ions during ferrite formation. The presence of Fe_2_O_3_ ions is confirmed by the satellite peak in the electronic spectra at 719 eV and the iron binding energy in Fe_2_O_3_, which is measured between 710 and 711 eV^85^.

The doublet peak of the O 1 s spectrum is seen in Fig. 15(d). At around 530 eV, the most powerful component is the oxygen in the oxide lattice (O_L_). The secondary peak, situated at about 532 eV, is associated with hydroxides and defective oxygen (O_V_)^86^. Surprisingly, the area ratio of O_L_/O_V_ decreased as x rose to 0.08, indicating a consistent increase in the concentration of oxygen vacancies. This increase is caused by the substitution of Al³⁺ ions, which can alter the spinel structure’s lattice stability and local charge balance. Al³⁺ with varying valence and site preference can cause charge compensation and mild lattice deformation, which might encourage the creation of oxygen vacancies.

Three satellite peaks at 861 eV, 866 eV, and 877 eV were recognized as Ni 2p_3/2_ and Ni 2p_1/2_ regions in the Ni 2p spectra, as shown in Fig. 15(e). These peaks suggested the presence of Ni^2+^ ions in both tetrahedral and octahedral positions. Two strong peaks representing the octahedral and tetrahedral coordination were obtained from the deconvolution of Ni 2p_3/2_ at 854.5 eV and 856.5 eV, respectively. During the deconvolution of Ni 2p_1/2_, two significant peaks were produced at 871.5 eV and 873.3 eV, respectively, indicating the octahedral and tetrahedral coordination^87^.

The characteristic binding energies in the XPS Al 2p spectra in Fig. 15(f), which are around 74–76 eV and correspond to oxidized Al in Al–O bonds rather than metallic Al, demonstrate that Al^3+^ was successfully integrated into the spinel ferrite lattice. The two components that deconvolution exposes show that Al^3+^ ions occupy both tetrahedral and octahedral sites; nevertheless, a greater preference for octahedral sites is expected because of Al^3+^’s lower ionic radius in octahedral coordination. A significant increase in peak intensity from 0.04 to 0.08 with increasing Al content supports higher degrees of substitution. However, a little widening suggests more local lattice deformation and cation redistribution. Because Al^3+^ substitution at octahedral sites can impact superexchange interactions, cation distribution, and ultimately the magnetic and electrical characteristics of ferrites, these site occupancies are significant^30^.

### Magnetic properties

#### VSM analysis

**Fig. 16:**
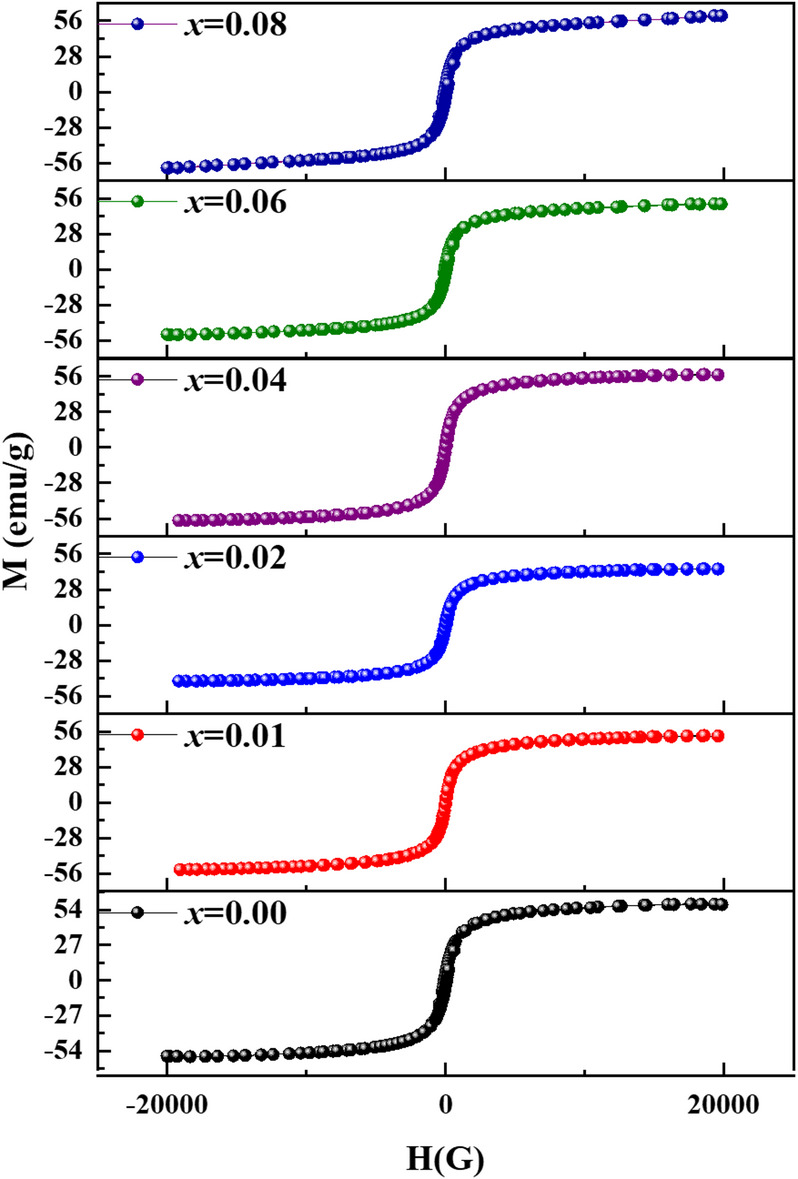
M–H hysteresis plots of Co_0.6−x_Zn_0.4−x_Ni_x_Al_x_Fe_2_O_4_ nanoparticles (0.00 ≤ *x* ≤ 0.08).

Figure 16 depicts the ambient temperature M-H hysteresis loops used to examine the impact of Ni^2+^ -Al^3+^ co-doping on the magnetic characteristics of Co_0.6−x_Zn_0.4−x_Ni_x_Al_x_Fe_2_O_4_ nanoparticles. Table 4 lists many significant magnetic parameters, including saturation magnetization (*M*_*s*_), remnant magnetization (*M*_*r*_), coercivity ($\text{}H_c\text{), and squareness ratio } SQR\left(\frac{M_r}{M_s}\right)$. The findings, in Table 4, show that when Ni^2+^ and Al^3+^ ions are introduced in to the Co_0.6−x_Zn_0.4−x_Ni_x_Al_x_Fe_2_O_4_ ferrite structure,$\:{\:M}_{s}$,$\:{\:M}_{r}$, and $\:{H}_{c}$ values decrease as x increases from 0.00 to 0.08. The observed drop was caused by Ni²⁺ ions (2 µB) and Al³⁺ ions (0 µB) partially replacing the Zn²⁺ ions (0 µB) and Co²⁺ ions (3 µB), respectively^88,89^. The total magnetic moment $\:{\mu\:}_{th}$ in spinel ferrites is determined by Néel’s two-sublattice model^89^ by subtracting the magnetic moments at the tetrahedral M_T_ and octahedral M_O_ sites as follows:

18 $$\:{\mu\:}_{th}\:={\:M}_{O}-{M}_{T}$$

The decrease in *M*_*s*_, and thus the net magnetization, is attributed to the dilution of octahedral sites caused by the incorporation of Ni²⁺ and Al³⁺ ions, leading to a reduction in the octahedral magnetic moment *M*_*O*_ ​. Table 4 shows that co-doping with Ni²⁺ and Al³⁺ results in a similar decrease in *M*_*r*_ (from 4.48 emu/g to 1.27 emu/g) and H_c_ (from 79.02 G to 20.95 G). XRD and TEM analyses confirm that the reduction in H_c_ and M_r_ is associated with the formation of smaller nanoparticles. Increased Ni²⁺-Al³⁺ co-doping reduces particle size and increases surface-to-volume ratio, resulting in magnetically disordered surface layers. This surface spin canting reduces the effective magnetic volume, causing discrepancies between theoretical and experimental magnetic moments. The observed reduction in *M*_*s*_ (58 → 51 emu g⁻¹) cannot be attributed solely to cation redistribution; surface spin disorder likely plays a significant role, especially at the smallest crystallite sizes. It is also clear, From Table 4, that Ni^2+^-Al^3+^ co-doped samples have lower SQR values than undoped sample, decreasing from 0.076 to 0.024 as x increases from 0.00 to 0.08; consequently, SQR values below 0.5 indicate the formation of single magnetic domain particles^90^. These effects collectively contribute to the evolution of reduced magnetic ordering behavior^90,91^. SQR value fluctuation is comparable to the variation of $\:{M}_{s}$, $\:{M}_{r}$,and $\:{H}_{c}$with x. Consequently, particle size and cation distribution have a significant impact on the magnetic characteristics of Co_0.6−x_Zn_0.4−x_Ni_x_Al_x_Fe_2_O_4_ nanoparticles.

**Table 4:** Magnetic parameters of Co_0.6−x_Zn_0.4−x_Ni_x_Al_x_Fe_2_O_4_ nanoparticles, including saturation magnetization (Mₛ), remnant magnetization (M_r_), coercivity (H_c_), and squareness ratio (SQR) as a function of Ni²⁺–Al³⁺ co-doping (x).

Sample (x)	$\:{\boldsymbol{M}}_{\boldsymbol{s}}\:$(emu/g)			$\:{\boldsymbol{M}}_{\boldsymbol{r}}$ (emu/g)	$\:{\boldsymbol{H}}_{\boldsymbol{c}}$ (G)	SQR
0.00	58.47			4.48	79.02	0.076
0.01	57.51			3.59	65.42	0.062
0.02	57.26			3.05	62.01	0.053
0.04	52.52			2.59	42.15	0.049
0.06	51.81			1.39	39.01	0.027
0.08	51.43			1.27	20.95	0.024

To contextualize these results, Table 5 compares the structural, magnetic, and optical properties of the present Co–Zn–Ni–Al nanoferrites with previously reported ferrites^90–92^. The crystallite size in this work (14–23 nm) is smaller than that reported for Ni–Zn ferrites (20–50 nm), Ni–Zn–Al ferrites (17–40 nm), and Zn–Co–Ni ferrites (15–60 nm)^90–92^. The reduced size increases the surface-to-volume ratio, which may induce mild surface spin canting. The lattice parameters of the present samples (8.366–8.374 Å) are slightly larger than those reported for similar Ni–Zn–Al ferrites (≈ 8.35–8.36 Å)^90,91^, likely due to the influence of Ni²⁺ and Al³⁺ substitution in the spinel lattice. Importantly, the cubic spinel structure remains unchanged, indicating stable incorporation of the co-dopants. Magnetically, the samples exhibit relatively high saturation magnetization (*M*_*s*_ = 51–58 emu/g) compared with Ni–Zn–Al ferrites (35–55 emu/g) and Zn–Co–Ni ferrites (30–60 emu/g), suggesting that A–B superexchange interactions remain largely preserved despite partial B-site substitution. The coercivity (*H*_*c*_ = 20–79 Oe) is lower than that reported for many ferrites, which can be attributed to Co dilution, small crystallite size, and moderate surface spin canting, all of which favor soft magnetic behavior. Overall, Ni²⁺–Al³⁺ co-doping in Co–Zn ferrites maintains ferromagnetic coupling, structural stability, and magnetic softness simultaneously. The combination of reduced crystallite size, slight lattice expansion, and improved magnetic properties highlights the potential of this system for multifunctional magnetic and optoelectronic applications.

**Table 5:** Comparison of structural, magnetic, optical, and lattice parameters of different ferrite systems with current study.

System	Synthesis	Crystallite size (nm)	Lattice Constant (Å)	M_s_ (emu/g)	H_c_ (Oe)	Magnetic Trend
Ni-Zn [90]	Microwave combustion	20–50	8.34–8.36	40–65	50–150	Ni lowers anisotropy
Ni-Zn-Al [91]	Hydrothermal	17–40	8.35–8.36	35–55	60–180	Al weakens A-B interaction
Zn-Co-Ni [92]	Sol–gel	15–60	8.36–8.37	30–60	30–200	Co controls anisotropy
Co-Zn-Ni-Al [current study]	coprecipitation	14–23	8.366–8.374	51–58	20–79	High M_s_ and low H_c_ (soft ferrite)

#### Magnetic data modeling

Numerous iterations of the law of approach to saturation (LAS) were studied in order to examine the relationship between magnetization and the applied magnetic field. To provide a more precise empirical connection, Akulov’s theory, which first explained the magnetization based on 1/H^2^ alone, was extended to include terms like 1/H, 1/H^2^, and H. Every prepared sample used in this study was subjected to trial fitting, with a defined upper limit and a fixed lower field limit of 0 G. The following models were evaluated^93^:

19 $$\:\mathrm{M}\mathrm{o}\mathrm{d}\mathrm{e}\mathrm{l}\:1\:\left(\mathrm{M}1\right):\mathrm{M}=\:{\mathrm{M}}_{\mathrm{s}\:}\left(1-\:\frac{\mathrm{b}}{{\mathrm{H}}^{2}}\right)$$

20 $$\:\mathrm{M}\mathrm{o}\mathrm{d}\mathrm{e}\mathrm{l}\:2\:\left(\mathrm{M}2\right):\mathrm{M}=\:{\mathrm{M}}_{\mathrm{s}\:}\left(1-\:\frac{\mathrm{a}}{\mathrm{H}}-\:\frac{\mathrm{b}}{{\mathrm{H}}^{2}}\right)$$

21 $$\:\mathrm{M}\mathrm{o}\mathrm{d}\mathrm{e}\mathrm{l}\:3\:\left(\mathrm{M}3\right):\mathrm{M}=\:{\mathrm{M}}_{\mathrm{s}\:}\left(1-\:\frac{\mathrm{a}}{\mathrm{H}}-\:\frac{\mathrm{b}}{{\mathrm{H}}^{2}}\right)+\:{\upchi\:}\mathrm{H}$$

22 $$\:\mathrm{M}\mathrm{o}\mathrm{d}\mathrm{e}\mathrm{l}\:4\:\left(\mathrm{M}4\right):\mathrm{M}=\:{\mathrm{M}}_{\mathrm{s}\:}\left(1-\:\frac{\mathrm{b}}{{\mathrm{H}}^{2}}\right)+\:{\upchi\:}\mathrm{H}$$

23 $$\:\mathrm{M}\mathrm{o}\mathrm{d}\mathrm{e}\mathrm{l}\:5\:\left(\mathrm{M}5\right):\mathrm{M}=\:{\mathrm{M}}_{\mathrm{s}\:}\left(1-\:\frac{1}{15}\times\:\:\frac{{\mathrm{H}}_{\mathrm{A}}^{2}}{{\mathrm{H}}^{1/2}\:({\mathrm{H}}^{3/2}+\:{\mathrm{H}}_{\mathrm{R}}^{3/2})}\right)+\:{\upchi\:}\mathrm{H}$$

**Fig. 17:**
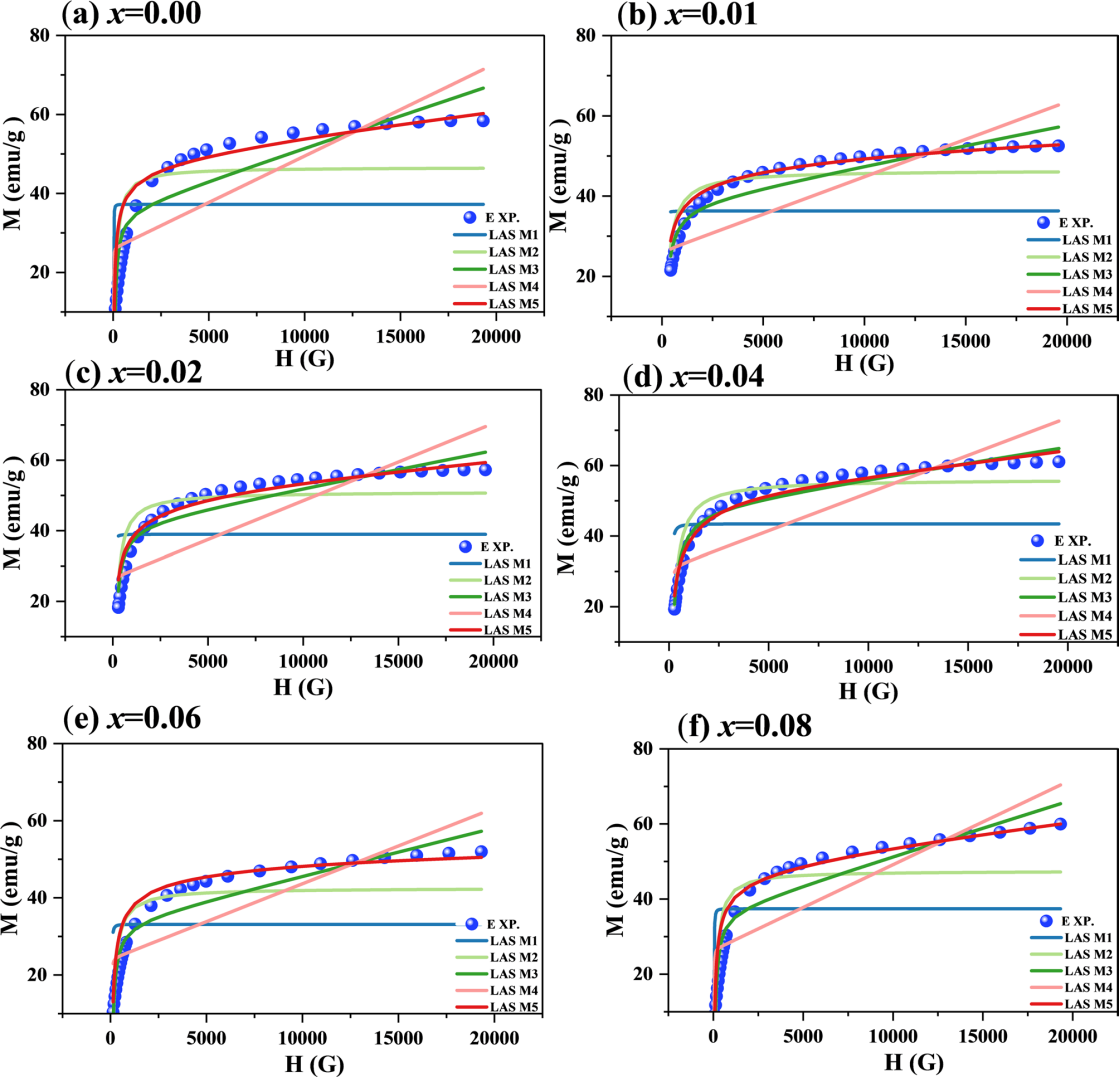
**(a-f)** LAS fitting for Co_0.6−x_Zn_0.4−x_Ni_x_Al_x_Fe_2_O_4_ nanoparticles (0.00 ≤ *x* ≤ 0.08).

In these models, each letter represents a distinct influencing factor: a/H represents magnetic hardness due to structural flaws, b/H^2^ represents magnetocrystalline anisotropy, and χH represents paramagnetic or antiferromagnetic contributions. A thorough connection that takes exchange, magnetic, and anisotropy fields into consideration is offered by M5^94^. The prepared samples’ LAS fittings are displayed in Fig. 17 (a-f).

**Fig. 18:**
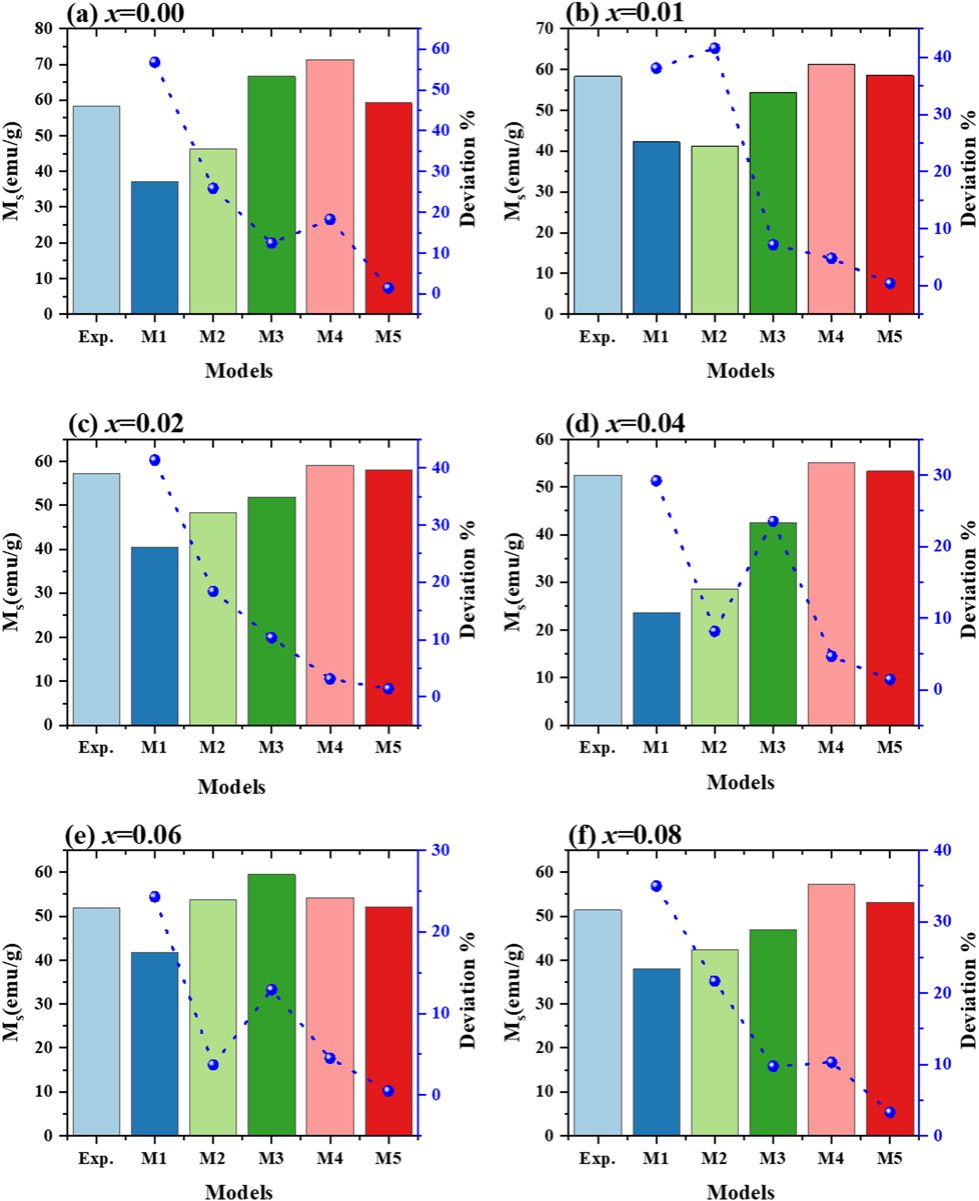
**(a-f)** Variation of M_s_ values with standard deviation for the law of approach to saturation models applied to Co_0.6−x_Zn_0.4−x_Ni_x_Al_x_Fe_2_O_4_ nanoparticles (0.00 ≤ *x* ≤ 0.08).

**Fig. 19:**
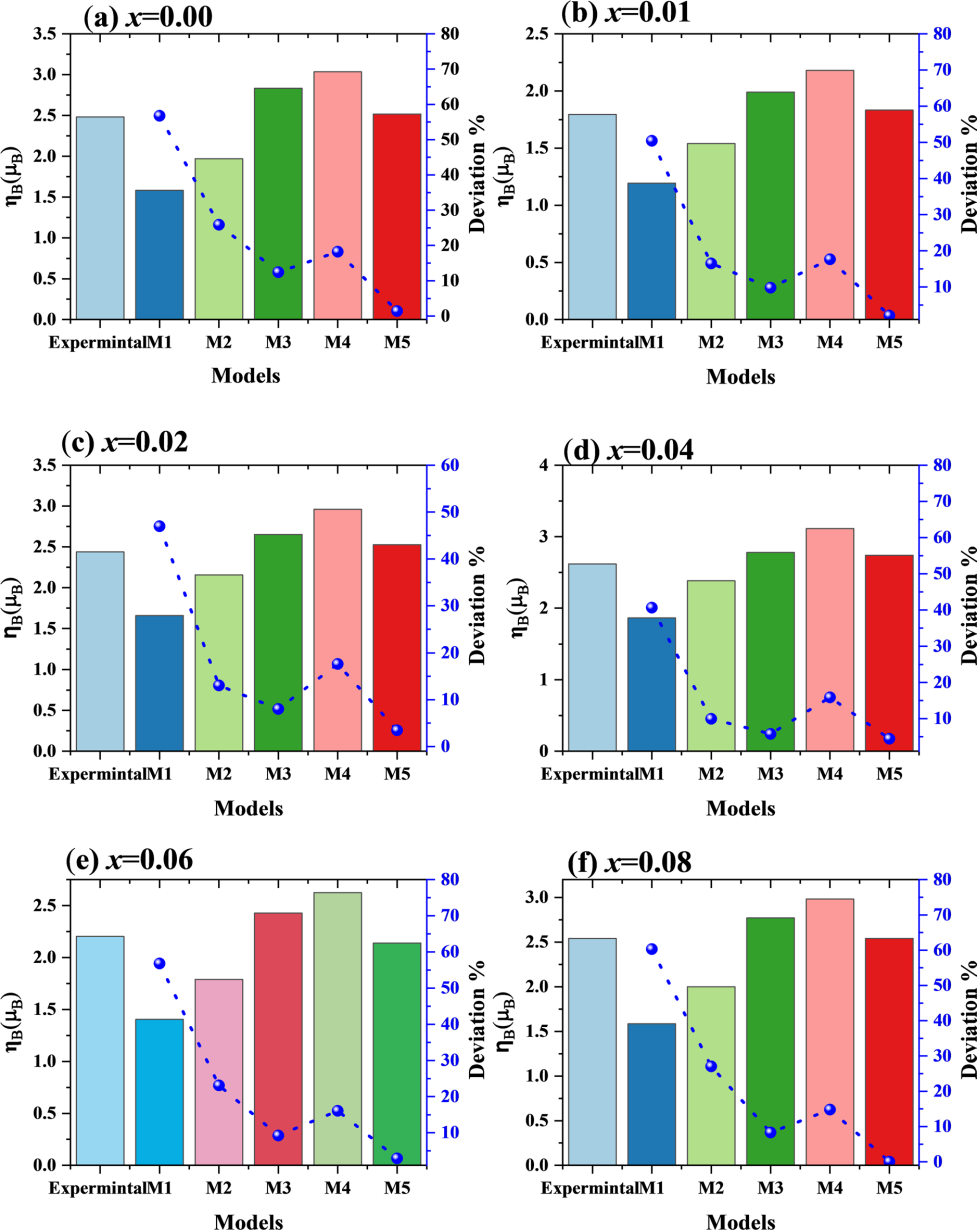
**(a-f)** Variation of *ɳ*_*B*_ values with standard deviation for the law of approach to saturation models applied to Co_0.6−x_Zn_0.4−x_Ni_x_Al_x_Fe_2_O_4_ nanoparticles (0.00 ≤ *x* ≤ 0.08).

The $\:{M}_{s}$ recovered values from Models 4 and 5 are similar to the experimental $\:{M}_{s}$ values, as illustrated in Fig. 18. This is evident from the low standard deviation values. The following formula was used to determine the experimental magnetic moment $\:{\eta}_{B}$^95^:

24 $$\eta_{B}=\frac{{M}_{w}{M}_{s}}{5585}$$

where the molecular weight is $\:{M}_{w}$, *ɳ*_*B*_ has a trend similar to the fluctuation in $\:{M}_{s}$, as illustrated in Fig. 19, which is to be expected given that *ɳ*_*B*_ is directly proportional to $\:{M}_{s}$. Nevertheless, as the high standard deviation indicates, the $\:{M}_{s}$ and *ɳ*_*B*_ values taken from Model 4 are not consistent with the experimental results. Table 5 summarizes the R^2^ values, extracted parameters, and fitting parameters from each model, respectively.

**Table 6:** Magnetic parameters extracted from the law of approach to saturation (LAS) models (Models 1–5) for Co_0.6−x_Zn_0.4−x_Ni_x_Al_x_Fe_2_O_4_ nanoparticles (0.00 ≤ x ≤ 0.08).

LAS Parameter	x					
	0	0.01	0.02	0.04	0.06	0.08
Model 1 $\:\mathrm{M}=\:{\mathrm{M}}_{\mathrm{s}\:}\left(1-\:\frac{\mathrm{b}}{{\mathrm{H}}^{2}}\right)$						
b (G^2^)	206.714	1149.38	1033.6	44883.9	1096.41	608.23
R²	0.829	0.875	0.844	0.878	0.854	0.845
Model 2 $\:\mathrm{M}=\:{\mathrm{M}}_{\mathrm{s}\:}\left(1-\:\frac{\mathrm{a}}{\mathrm{H}}-\:\frac{\mathrm{b}}{{\mathrm{H}}^{2}}\right)$						
a (G)	109.41	189.38	165.79	214.02	151.95	131.94
b (G^2^)	−1453.92	−5365.22	−4330.78	−10321.3	−4125.31	−2810.76
R^2^	0.936	0.976	0.976	0.987	0.959	0.952
Model 3 $\:\mathrm{M}=\:{\mathrm{M}}_{\mathrm{s}\:}\left(1-\:\frac{\mathrm{a}}{\mathrm{H}}-\:\frac{\mathrm{b}}{{\mathrm{H}}^{2}}\right)+\:{\upchi\:}\mathrm{H}$						
a (G)	91.88	161.05	147.99	191.06	126.58	109.2
b (G^2^)	−1196.56	−4417.63	−3783.49	−8874.12	−3301.02	−2251.28
χ × 10^− 4^ (emu. g^− 1^.G^− 1^)	16.25	10.11	10.61	8.92	12.37	15.02
R²	0.978	0.992	0.991	0.995	0.988	0.986
Model 4 $\:\mathrm{M}=\:{\mathrm{M}}_{\mathrm{s}\:}\left(1-\:\frac{\mathrm{b}}{{\mathrm{H}}^{2}}\right)+\:{\upchi\:}\mathrm{H}$						
b (G^2^)	189.41	1075.87	940.81	4200.13	991.59	545.61
χ × 10^− 4^ (emu.g^− 1^.G^− 1^)	23.52	18.66	21.97	21.36	19.01	22.71
R^2^	0.945	0.961	0.948	0.96	0.956	0.955
Model 5 $\:\mathrm{M}=\:{\mathrm{M}}_{\mathrm{s}\:}\left(1-\:\frac{1}{15}\times\:\:\frac{{\mathrm{H}}_{\mathrm{A}}^{2}}{{\mathrm{H}}^{1/2}\:({\mathrm{H}}^{3/2}+\:{\mathrm{H}}_{\mathrm{R}}^{3/2})}\right)+\:{\upchi\:}\mathrm{H}$						
H_A_ ×10^4^ (G)	2.21	1.98	1.97	1.57	1.5	1.41
H_R_ ×10^4^ (G)	3.04	1.92	1.83	1.32	1.25	1.2
χ × 10^− 4^ (emu.g^− 1^.G^− 1^)	0.61	2.03	5.11	7.72	8.77	9.72
K (×10^4^ erg. g^− 1^)	64.51	57.74	56.41	41.23	38.85	36.26
R^2^	0.984	0.981	0.987	0.994	0.981	0.981

For Co_0.6−x_Zn_0.4−x_Ni_x_Al_x_Fe_2_O_4_, model 5 (M5), which includes exchange–correlation fields and magnetocrystalline anisotropy, provided the most reliable description among the five examined models. As shown in Table 6, M5 yields consistent anisotropic fields (H_A_ and H_R_), positive b values, and a high coefficient of determination (R² ≈ 0.98) for all compositions. Although the simpler models (M2 and M3) produced acceptable statistical fits, they frequently resulted in unphysical negative b values, while M1 failed to adequately describe the data. This indicates that additional factors such as structural defects, porosity, and anisotropies contribute to the magnetization behavior beyond the 1/H² term. The fitted values of *M*_*s*_ and ɳ_B_ (Figs. 18 and 19) further confirm the reliability of M5, whereas M4 tends to underestimate *M*_*s*_ and shows larger fluctuations in ɳ_B_. The observed decrease in *M*_*s*_ and ɳ_B_ with increasing *x* is attributed to Ni²⁺/Al³⁺-induced cation redistribution, where these ions preferentially occupy octahedral sites, altering the magnetic interactions. Overall, the LAS analysis indicates that M5 is the most appropriate model for describing the high-field magnetization behavior of the prepared spinel ferrites.

The anisotropy field $\:{H}_{A}$ decreased erratically; as *x* increased from 0.00 to 0.08, it decreased from 2.21 × 10^4^ to 1.41 × 10^4^ G. The following formula was used to determine the local magnetocrystalline anisotropy (K)^94^:

25 $$\:K=\frac{{H}_{A}\:\times\:\:{M}_{s}}{2}.$$

The decrease in $\:{M}_{s}$is consistent with the anisotropy constant K values listed in Table 6. The LAS model (M5) shows lower K values (from 57.74 × 10^4^ to 36.26 × 10^4^ erg.g^− 1^) compared with the pure nanoparticles (64.51 × 10^4^ erg.g^− 1^), confirming the formation of a mixed spinel after Ni²⁺–Al³⁺ co-doping. The reduction in magnetocrystalline anisotropy is mainly due to the dilution of Co²⁺ ions at the octahedral (B) sites. Since anisotropy in Co-based spinels is largely governed by the orbital contribution of Co²⁺ and strong spin–orbit coupling^92^, substitution with Ni²⁺ and non-magnetic Al³⁺ weakens this interaction and lowers KKK. In addition, Al³⁺ at B-sites disturbs the A–B superexchange interaction, further reducing the anisotropy^96^. However, the decrease in Co²⁺ content remains the dominant factor.

### Cation distribution and magneto-structural correlation

The cation distribution of Co_0.6−x_Zn_0.4−x_Ni_x_Al_x_Fe_2_O_4_ nanoparticles (x = 0, 0.04, and 0.08) was estimated using a combined study of XPS deconvolution, Raman vibrational evolution (A₁g mode), and magnetic data interpreted within Néel’s two-sublattice model (refer to Table 7). Such multi-technique correlation is commonly used in spinel ferrites to address site occupancy and exchange interactions^90–92^. The distribution was built with stringent crystallographic restrictions: the tetrahedral (A) site includes Zn²⁺, Co²⁺, Ni²⁺, Fe²⁺, and Fe³⁺, whereas the octahedral (B) site only contains Co²⁺, Ni²⁺, Al³⁺, Fe²⁺, and Fe³⁺. For x = 0, Ni²⁺ and Al³⁺ are not present, which is consistent with the nominal composition. Such site-selective substitution and its impact on structural and functional features have been extensively studied in complex oxide systems, including A- and B-site substituted perovskites^97^. All proposed distributions meet the stoichiometric balance, charge neutrality, and total cation requirement of three per formula unit. For the undoped composition (x = 0), the expected cation arrangement is:

26 $$(\mathrm{Co}^{2+}_{0.15}\mathrm{Zn}^{2+}_{0.40}\mathrm{Fe}^{3+}_{0.40}\mathrm{Fe}^{2+}_{0.05})^{A}[\mathrm{Co}^{2+}_{0.45}\mathrm{Fe}^{3+}_{1.40}\mathrm{Fe}^{2+}_{0.15}]^{B}\mathrm{O}_{4}$$

This arrangement shows significant Fe³⁺ occupancy on both sublattices, with a humble Fe²⁺ proportion, which is compatible with XPS quantification and earlier results on Ni-Zn and Co-based ferrites^90,92^. Sublattice magnetic moments are determined to be M_A_= 2.65 µ_B_​ and M_B_= 8.95 µ_B_, resulting in a theoretical magnetic moment of µ_th_ = 6.30 µB per formula unit using the Néel’s two-sublattice model equation µ_th_=∣M_B_−M_A_∣. The experimentally calculated magnetic moment, computed from $\:{{{\upmu\:}}_{\mathrm{B}}}^{\mathrm{e}}=\frac{{\mathrm{M}}_{\mathrm{w}}{\mathrm{M}}_{\mathrm{s}}}{5585}$ is 2.48 µB. This indicates strong spin non-collinearity and surface canting typical of nanoscale ferrites^90,91^. Upon substitution (x = 0.04), the distribution changes into:

27 $$(\mathrm{Co}^{2+}_{0.16}\mathrm{Zn}^{2+}_{0.36}\mathrm{Ni}^{2+}_{0.02}\mathrm{Fe}^{3+}_{0.41}\mathrm{Fe}^{2+}_{0.05})^{A}[\mathrm{Co}^{2+}_{0.40}\mathrm{Ni}^{2+}_{0.02}\mathrm{Al}^{3+}_{0.04}\mathrm{Fe}^{3+}_{1.37}\mathrm{Fe}^{2+}_{0.17}]^{B}\mathrm{O}_{4}$$

The addition of Ni²⁺ and Al³⁺ causes significant redistribution of Fe²⁺/Fe³⁺ between sublattices while preserving overall charge balance. Al³⁺ preferentially occupies the octahedral site, resulting in partial magnetic dilution of the B sublattice, similar to findings in Al-substituted spinel ferrites^91^. In Ni-Zn-Co ferrite systems, a percentage of Co²⁺ migrates to the A site, lowering magnetic contrast across sublattices^92^. The estimated magnetic moment drops to µth = 5.94 µB, whereas the observed value reduces to 2.24 µB, indicating weakening of A-B superexchange interaction. Further substitution (x = 0.08) yields the distribution:

28 $$(\mathrm{Co}^{2+}_{0.18}\mathrm{Zn}^{2+}_{0.32}\mathrm{Ni}^{2+}_{0.04}\mathrm{Fe}^{3+}_{0.38}\mathrm{Fe}^{2+}_{0.08})^{A}[\mathrm{Co}^{2+}_{0.34}\mathrm{Ni}^{2+}_{0.04}\mathrm{Al}^{3+}_{0.08}\mathrm{Fe}^{3+}_{1.42}\mathrm{Fe}^{2+}_{0.12}]^{B}\mathrm{O}_{4}$$

The rising concentration of Al³⁺ on the B site accelerates octahedral magnetic dilution, while Ni²⁺ (2 µB) gradually replaces the higher-moment Co²⁺ (3 µB). The Fe²⁺ percentage increases somewhat, altering the Fe-O-Fe superexchange routes and increasing magnetic frustration. Similar magnetic suppression tendencies have been seen in Ni-Zn-Al ferrites at nano- to microscale levels^91^. The predicted magnetic moment reduces to 5.28 µB, but the measured result is 2.21 µB. The rising gap between theoretical and experimental moments implies increased spin canting and surface disorder caused by replacement^90,91^.

Raman A₁g mode development confirms this hypothesis. The A₁g vibration involves symmetric stretching of tetrahedral metal-oxygen bonds and is extremely sensitive to cation occupancy at the A location. The progression of the A₁g intensity ratio (1.87 → 1.88 → 5.12) implies tetrahedral deformation and redistribution of magnetic cations, which aligns with the improved cation model. Such structural distortion promotes non-collinear spin alignment and reduces net magnetisation. Similar substitution-driven lattice distortion and property development have been seen in doped oxide systems where cation engineering changes the local bonding microenvironment^98,99^.

The magnetic suppression observed over the series is driven by a cooperative process involving octahedral dilution by non-magnetic Al³⁺, substitution of high-moment Co²⁺ with lower-moment Ni²⁺, partial migration of Co²⁺ to tetrahedral locations, and modest redistribution of Fe²⁺/Fe³⁺. While the drop in theoretical moment is due to intrinsic sublattice alteration, the more significant reduction in actual magnetisation indicates improved nanoscale spin canting and exchange competition. The remarkable association between XPS-derived valence states, Raman structural signals, and magnetic measurements allows for a coherent and internally consistent explanation of the magnetostructural development in this spinel system.

**Table 7:** Theoretical moment, experimental momnet, Raman A_1g_ mode ratio, and Cation distribution for Co_0.6−x_Zn_0.4−x_Ni_x_Al_x_Fe_2_O_4_ ferrites with x = 0, 0.04, and 0.08.

x	µ_th_ (µ_B_)	$\:{{\boldsymbol{\upmu\:}}_{\mathbf{B}}}^{\mathbf{e}}$ (μ_B_)	A_₁g_(1)/A_₁g_(2) ratio	Cation Distribution
0.00	6.30	2.48	1.87	(Co^2+^_0.15_Zn^2+^_0.40_Fe³⁺_0.40_Fe²⁺_0.05_) ^A^[Co^2+^_0.45_Fe³⁺_1.40_Fe²⁺_0.15_]^B^ O₄
0.04	5.94	2.24	1.88	(Co^2+^_0.16_ Zn^2+^_0.36_Ni^2+^_0.02_Fe³⁺_0.41_Fe²⁺_0.05_)^A^[Co^2+^_0.40_Ni^2+^_0.02_Al^3+^_0.04_Fe³⁺_1.37_Fe²⁺_0.17_]^B^ O₄
0.08	5.40	2.21	5.12	(Co^2+^_0.18_ Zn^2+^_0.32_Ni^2+^_0.04_Fe³⁺_0.38_Fe²⁺_0.08_)^A^[Co^2+^_0.34_Ni^2+^_0.04_Al^3+^_0.08_Fe³⁺_1.42_Fe²⁺_0.12_]^B^ O₄

## Conclusions

This study effectively synthesized Co_0.6−x_Zn_0.4−x_Ni_x_Al_x_Fe_2_O_4_ nanoferrites (0.00 ≤ x ≤ 0.08) using the co-precipitation technique. Structural investigation verified the production of single-phase cubic spinel structures in all compositions. Increasing Ni^2+^-Al^3+^ concentration led to a small rise in lattice parameter (8.366–8.374 Å) due to cation redistribution and valence compensation. The XPS results confirmed the conversion of Fe³⁺ to Fe²⁺ ions and the redistribution of cations between tetrahedral (A) and octahedral (B) sites to preserve the charge neutrality. The spinel structure was preserved with distinctive metal-oxygen vibrations, as validated by FTIR and Raman spectra. SEM pictures revealed almost spherical nanoparticles with little aggregation, while EDX analysis verified good purity and stoichiometric composition. Optical studies revealed a blue shift in the absorption edge with increasing co-doping level. The direct and indirect band gap energies increased from 3.22 to 3.32 eV and 2.75 to 2.88 eV, respectively, whereas the Urbach energy fell (0.335 − 0.262 eV), suggesting more crystallinity and lower structural disorder. Magnetic investigations demonstrated a tendency toward reduced magnetic ordering, with decreasing coercivity and remanence as particle size dropped. LAS analysis using the M5 model yielded high fitting accuracy (R² = 0.98). The anisotropy field (H_A_) and anisotropy constant (K) decreased in a systematic manner with increasing Ni^2+^-Al^3+^ substitution, indicating weaker magnetocrystalline anisotropy and increased magnetic softness. Ni²⁺-Al³⁺ co-doping improves structural, optical, and magnetic characteristics via controlling cation redistribution and anisotropy modulation. These nanoferrites’ multifunctional properties make them interesting candidates for advanced magnetic and optoelectronic applications, such as magnetic hyperthermia and spintronic devices.

## Data Availability

The data that support the findings of this study are available from the corresponding author upon reasonable request.
